# Tumor-neuroglia interaction promotes pancreatic cancer metastasis

**DOI:** 10.7150/thno.42440

**Published:** 2020-04-06

**Authors:** Dan Su, Xiaofeng Guo, Leyi Huang, Huilin Ye, Zhiguo Li, Longfa Lin, Rufu Chen, Quanbo Zhou

**Affiliations:** 1 Guangdong Provincial Key Laboratory of Malignant Tumor Epigenetics and Gene Regulation, Sun Yat-sen Memorial Hospital, Sun Yat-sen University, Guangzhou, Guangdong Province, China; 2 Department of Pancreatobiliary Surgery, Sun Yat-sen Memorial Hospital, Sun Yat-sen University, Guangzhou, Guangdong Province, China; 3 Department of General Surgery, Guangdong Provincial People's Hospital, Guangdong Academy of Medical Sciences, Guangzhou, Guangdong Province, China

**Keywords:** pancreatic ductal adenocarcinoma, Schwann cell, epithelial-mesenchymal transition, metastasis, interaction

## Abstract

**Rationale**: The peripheral nervous system (PNS) plays an important role in tumor growth and progression. Schwann cells (SCs), the main glia cells of the PNS, augment cancer metastasis in contact-dependent or contact-independent manner in various malignancies. In the present study, we aimed to determine whether interplay between pancreatic cancer cells and SCs via paracrine signaling contributes to cancer progression.

**Methods**: Immunofluorescence analysis was performed to reveal the distribution of SCs in PDAC tissues and to determine the prognostic value and clinicopathological relevance of the level of intra‑tumoral SC markers for patients diagnosed with PDAC. Transwell assays and wound healing assays were carried out to investigate the influence of SC conditioned medium (SCM), SC co‑culture, or co-cultured CM on the migratory and invasive abilities of pancreatic cancer cells. The mechanism of SCs induced cancer cells migration and invasion was confirmed using quantitative real-time reverse transcription polymerase chain reaction (qRT-PCR), enzyme-linked immunosorbent assays (ELISAs), western blotting, immunofluorescence, immunohistochemistry, siRNA-mediated gene interference, and an* in vivo* mouse model.

**Results**: Immunofluorescence analysis of tissue samples revealed that there were two different types of SCs distributed in the tumor microenvironment, the presence of which correlated with several clinicopathological characteristics and overall survival for patients with PDAC. Although SCM had no impact on the motility and invasiveness of tumor cells, both co-cultivation with SCs and co‑cultured CM enhanced pancreatic cancer cell migration and invasion. Mechanistically, SC‑derived Interleukin 6 (IL6), which was induced by co-culture with pancreatic cancer cells, augmented cancer cell migration and invasion by activating STAT3 signaling in cancer cells, while IL6 neutralization or STAT3 downregulation abrogated these effects. Furthermore, Interleukin 1β (IL1β), secreted by tumor cells, activated the nuclear actor (NF)-kappa B pathway in SCs, resulting in increased cytokines production, including IL6, while inhibiting the IL1β-IL1R1 axis led to inactivation of NF-kappa B signaling and downregulated cytokines expression in SCs. Interfering with tumor-neuroglia crosstalk impeded cancer cell dissemination *in vivo*.

**Conclusion**: Schwann cells were extensively distributed in the PDAC tumor microenvironment and high level of intra-tumoral SC markers could serve as an independent prognostic factor for poor survival of patients with PDAC. The tumor-neuroglia interaction is indispensable for SCs to acquire a tumor-facilitating phenotype. Targeting the tumor-neuroglia interplay might be a promising strategy to treat PDAC.

## Introduction

Pancreatic ductal adenocarcinoma (PDAC) is ranked as the 14^th^ most common cancer and is the 7^th^ highest cause of cancer-related mortality in the world 1. By 2030, it is estimated that pancreatic cancer (PCa) will become the 2^nd^ most common cause of cancer-associated death in the United States 2,3. In spite of the rapid improvements in surgical techniques, chemotherapy medication, and the introduction of neo-adjuvant chemoradiotherapy, the long-term survival for patients suffering from PCa remains poor 1, mainly because of late diagnosis at an advanced stage with local invasion or distal metastasis 4. An important characteristic of PDAC is the excessively dense desmoplastic tumor microenvironment (TME), which comprises cellular and acellular components, such as fibroblasts, immune cells, and extracellular matrix (ECM). Accumulating studies have suggested that cells in the TME can work together to promote tumor initiation and progression 5,6. As a result, targeting the TME might lead to the development of novel effective therapeutic reagents for PDAC treatment.

Among the components of the TME, Schwann cells (SCs), which are a major component of the peripheral nervous system (PNS), play an important role in nerve regeneration 7. Recent studies showed that SCs actively participate in many aspects of tumor progression, including metastasis 8,9, perineural invasion (PNI) 10,11, PDAC-associated neural remodeling (PANR) 12, and immunomodulation 13, rather than just being bystanders. Nevertheless, the mechanism by which SCs facilitate tumor cells invasion and metastasis remains controversial. For instance, Deborde et al. reported that SCs induce pancreatic cancer cell invasion in a contact-dependent manner 11, while Na'ara et al. showed that SCs promote PNI through paracrine signaling 10. Therefore, further studies are urgently needed to investigate the interactions between SCs and tumor cells, as well as their roles in tumor progression.

Epithelial-mesenchymal transition (EMT), a process by which epithelial cells lose cell-to-cell junctions and acquire increased motility, contributes to the invasion and progression of tumors of epithelial origin 14. Recent studies showed that EMT is involved in SC-induced cancer metastasis in several solid malignancies, including salivary adenoid cystic carcinoma 9 and lung cancer 8. For example, Zhou at al. demonstrated that SCs conditioned by lung cancer cell secreted CXCL5 to activate PI3K/Akt/GSK-3β signaling and induced the expression of EMT regulators Snail and Twist in tumor cells, thereby aiding the spread and metastasis of lung cancer cells 8. Nevertheless, it remains to be investigated whether EMT is involved in SC-induced pancreatic cancer cell dissemination.

In the present study, we demonstrated that SCs are widely distributed in PDAC stroma. In addition, high level of intra-tumoral SC markers correlated significantly with an increased incidence of distant metastases, vascular invasion, and PNI, and predicted a dismal five-year survival rate for patients with PDAC. *In vitro* functional experiments showed that tumor-conditioned SCs acquire an immature phenotype, characterized by upregulated expression of a series of cytokines, thus promoting pancreatic cancer cells migration and invasion. Mechanistically, cancer cells secret interleukin 1β (IL1β) to activate the nuclear factor kappa B (NF-κB)/p65 pathway in SCs, thereby increasing the production of interleukin 6 (IL6) from SCs. In return, elevated IL6 in cell supernatants induces EMT, and the invasion and metastasis of tumor cells via signal transducer and activator of transcription 3 (STAT3) signaling. Taken together, our study revealed a non‑negligible role of the tumor-neuroglia interaction in tumor progression, and identified SCs as an active participant in addition to their roles in PNI. Further studies are necessary to develop novel treatment modalities targeting this underestimated element of the TME.

## Materials and Methods

### Patients and clinical samples

A total of 80 tissue samples were obtained from patients diagnosed with PDAC who underwent surgical resection in our department from May 2010 to April 2018. All patients provided informed consent for tissue collection, and our study was approved by the Ethical Committee of Sun Yat-sen University. The detailed clinicopathological characteristics of the enrolled patients are summarized in **Table 1**. The pathological tumor-node-metastasis (TNM) status was determined using the 8^th^ edition of the TNM classification of the American Joint Commission on Cancer. The overall survival time for each patient was defined as the time interval between the date of surgery and the date of death or the last day of follow-up.

### Cell lines and cell culture

Human pancreatic adenocarcinoma cell lines (AsPC-1, MIA PaCa-2), human SCs sNF96.2 and rat SCs RSC96 were purchased from the ATCC (Rockville, MD, USA). Cells were maintained in complete Dulbecco's modified Eagle's medium (DMEM, Biological Industries, Beit Haemek, Israel) or Roswell Park Memorial Institute (RPMI) 1640 medium (Biological Industries), supplemented with 10% fetal bovine serum (FBS, Biological Industries) and 1% penicillin/streptomycin (Biological Industries). Cells were cultured in a humidified incubator containing 5% CO_2_ at 37 °C.

### Drugs and reagents

The drugs and reagents used in this study comprised: Inhibitor of NF-κ kinase subunit beta (IKKβ) inhibitor ML120B (MedChemExpress, Monmouth Junction, NJ, USA), anti-human IL6 neutralizing antibody (R&D Systems, Minneapolis, MN, USA), anti-human IL1β neutralizing antibody (Abcam, Cambridge, MA, USA), recombinant human IL1β (Peprotech, Rocky Hill, NJ, USA), and recombinant human tumor necrosis factor alpha (TNFα) (Peprotech).

### Cell co-culture and conditioned medium preparation

To establish an *in vitro* co-culture system between tumor cells and human SCs (hSCs), 5 × 10^5^ hSCs were seeded at the bottom of six-well plates, while 5 × 10^5^ tumor cells (AsPC-1, MIA PaCa-2) were added into the upper Transwell insert (0.4 μm pore size, Corning, Inc., Corning, NY, USA). The co-culture system was maintained with complete medium for 24h or 48h for RNA extraction or co-cultured conditioned medium (co-CM) collection, respectively.

For the collection of SC conditioned medium (SCM), 6 × 10^5^ hSCs, or 1.2 × 10^6^ rat SCs (RSCs) were seeded in a six-well plate and allowed to attach overnight. The cells were then cultured in complete DMEM medium for 48 h to produce SCM. To prepare tumor conditioned medium (TCM), the culture medium of human pancreatic adenocarcinoma cells (AsPC-1, MIA PaCa-2) was replaced with complete medium when the cells reached an approximately 80% to 90% confluence. After 48 h of incubation, the supernatant was collected.

All the conditioned media were centrifuged at 2000 × *g* for 10 min to remove cells and cell debris, and then used immediately or frozen at -80 °C after allocation.

### RNA extraction and quantitative real-time reverse transcription PCR (qRT-PCR)

Total RNA from the cells lines was isolated using the Trizol reagent (Takara Bio, Inc., Shiga, Japan) according to the manufacturer's instructions. Total RNA (1 μg) was reverse transcribed using PrimeScript™ RT Master Mix (Takara Bio). Quantitative real-time polymerase chain reaction (qPCR) was performed using the TB Green™ Premix Ex Taq™ II (Tli RNaseH Plus, Takara Bio) and a Roche LightCycler 480 system (Roche, Basel, Switzerland). Thermal cycling of the qPCR reaction was started with a pre-denaturation step at 95 °C for 30 s and followed by 40 cycles of amplification (denaturation at 95 °C for 5 s, annealing and extension at 60 °C for 35s). All primers used for qRT-PCR are listed in **Table S1**. Gene mRNA levels were normalized using the expression of housekeeping gene *GAPDH* (encoding glyceraldehyde-3-phosphate dehydrogenase) and the relative fold changes were calculated using the 2^rr^CT method.

### Western blotting

Whole-cell extracts from cultured cells were obtained via cell lysis using lysis buffer plus protease- and phosphatase-inhibitors (1:100, 1:100 respectively) for 30 min on ice, followed by centrifugation at 12000 × *g* for 20 min at 4 °C. Proteins (20-40 μg) were separated using sodium dodecyl sulfate polyacrylamide gel electrophoresis (SDS-PAGE), transferred to polyvinylidene fluoride (PVDF) membranes, blocked with 5% bovine serum albumin (BSA in Tris-buffered saline-Tween 20 (TBST)), incubated with primary antibodies and then with horseradish peroxidase (HRP)‑linked secondary antibodies, followed by detection with an enhanced chemiluminescence (ECL) detection system. Images were quantified by densitometry using ImageJ software (National Institutes of Health (NIH), Bethesda, MD, USA) and processed using Adobe Photoshop CC2018 (Adobe, San Jose, CA, USA).

### Immunofluorescence analysis of human specimens

Formalin-fixed, paraffin-embedded tissue slides were deparaffinized in xylene, and then hydrated in a series of graded alcohol. The antigen retrieval procedure was performed using a pressure cooker for 15 min in 10 mM sodium citrate buffer (pH 6.0). The sections were then permeabilized in 0.5% Triton X-100/phosphate-buffered saline (PBS) for 10 min, blocked in 5% donkey serum/PBS (containing 0.5% Triton X-100) for 1 h, and incubated with primary antibodies against rabbit glial fibrillary acidic protein (GFAP) (1:400, Dako, Carpinteria, CA, USA), goat GFAP (1:100, Abcam), rabbit S100 (1:100, Abcam), mouse S100 Calcium Binding Protein B (S100) (1:50, Abcam), mouse aldehyde dehydrogenase 1 family member L1 (ALDH1L1) (1:40, Abcam), IL6 (1:50, Abcam) diluted in 5% donkey serum/PBS overnight at 4 °C. Fluorescent secondary antibodies, donkey anti-mouse Alexa Fluor 488 (1:300, Abcam), donkey anti-rabbit Alexa Fluor 555 (1:300, Abcam), and donkey anti-mouse Alexa Fluor 647 (1:300, Abcam), were used for secondary staining. Slides were mounted in 2-(4-amidinophenyl)-1H-indole-6-carboxamidine (DAPI) containing Vectashield mounting medium (Beyotime, Shanghai, China).

For the quantitative assessment of intra-tumoral SC density, five representative regions (the most abundant fields) from every section were photographed at 200 × magnification using a Leica DM2000 fluorescent microscope (Leica, Wetzlar, Germany). Schwann cells in the PDAC specimens were classified as intra-nerve SCs (within the pancreatic nerves) and stromal SCs (scattered in the PDAC stroma). The number of SCs, double immunolabeled with the glial makers GFAP and S100, was counted in each image by two observers independently. Subsequently, the number of total SCs for each patient was calculated using the arithmetic mean of the number of total SCs in the five representative regions. Finally, each patient was divided into a high or low intra-tumoral SCs density group according to the median value of total SCs.

### Immunohistochemistry (IHC)

Formalin-fixed, paraffin-embedded tissues were deparaffinized in xylene, and then hydrated in a series of graded alcohol. The antigen retrieval procedure was performed using a pressure cooker for 15 min in 10 mM sodium citrate buffer (pH 6.0). The sections were incubated with primary antibodies against IL6 (1:200, Abcam), phosphorylated (p)-NF-κB/p65 (S536) (1:100, Abcam) and S100 (1:500, Abcam) overnight at 4 °C. A REAL EnVision Detection Kit (HRP-linked, Dako) was used to reveal the locations of the antigens and nuclei were counterstained using hematoxylin.

To quantitatively assess the levels of IL6 and p-NF-κB/p65 in SCs, five representative regions (the strongest staining fields) from every section were photographed at 200 × magnification using a Nikon NI-U digital camera (Nikon, Tokyo, Japan). The IHC staining scores were calculated according to the following formula: IHC score = staining intensity (0, no staining; 1, light brown; 2, brown; 3, dark brown) × proportion of positively stained cells (0, none; 1, < 25%; 2, 25-50%; 3, 50-75%; and 4, > 75%). All samples were scored by two independent observers in a blinded manner.

### Immunocytometry/Immunofluresence

For immunofluorescence analysis, cells were fixed in 4% formaldehyde at room temperature for 20 min, permeabilized in 0.5% Triton X-100 at room temperature for 20 min, and blocked with 1% BSA/PBS at room temperature for 1 h. After incubation with primary antibodies against with p‑STAT3 (1:100, Cell Signaling Technology, Danvers, MA, USA) overnight at 4 °C, followed by incubation with secondary antibodies (donkey anti-rabbit Alexa Fluor 555 [1:300, Abcam]) in the dark for 1 h, cell nuclei were stained by incubation with DAPI at room temperature for 10 min. Images were obtained using a laser scanning confocal microscope (LSM800, Zeiss, San Diego, CA, USA).

### Transwell migration and invasion assays

Transwell migration and invasion assays of pancreatic cancer cells were performed to evaluate the effect of SCM, SC co-culture, or co-CM on the migratory and invasive abilities of pancreatic cancer cells in 8-μm 24-well Boyden chambers (Falcon, BD Biosciences, San Jose, CA, USA). For the invasion assays, Matrigel Basement Membrane Matrix (BD Biosciences) diluted with FBS‑free DMEM at a 1:9 ratio was precoated in the upper surface of the Transwell inserts and solidified for 30 min at 37 °C. Pancreatic cancer cells (1 × 10^5^ ) suspended in 200 μL of FBS-free DMEM were added to the upper chamber, while 500 μL of DMEM containing 1% FBS (control group), 450 μL SCM plus 50 μL DMEM containing 10% FBS (SCM group), 450 μL co-CM plus 50 μL DMEM containing 10% FBS (co-CM group), or SCs (1 × 10^5^ hSCs, 3 × 10^5^ RSC96, seeded into the lower chamber one day before the Transwell assays, SC group) plus 500 μL DMEM containing 1% FBS were added to the bottom chamber. The cancer cells were allowed to migrate or invade for 16 h and 48 h, respectively. Non-migrated and non-invaded cells were removed from the upper surface of the chamber, while the migratory and invasive cells were fixed in 4% formaldehyde at room temperature for 20 min and then stained with 0.1% crystal violet at room temperature for 20 min. Images were captured using a Nikon NI-U digital camera (Japan) and migratory and invasive cells were counted in five representative microscopy fields per chamber using ImageJ software (NIH). Experiments were performed in triplicate.

### Wound healing assay

Pancreatic cancer cells (AsPC-1, MIA PaCa-2) were seeded in six-well plates and scratched using a 200 μL sterile pipette tip when cells reached approximately 90% confluence. After washing with sterile PBS three times to remove the detached cells, AsPC-1 or MIA PaCa-2 cells were cultured in medium plus 1% FBS (control group), SCM plus 1% FBS (SCM group), co-cultured CM plus 1% FBS (co-cultured CM group) for 24 h or 48 h, respectively. Especially, for the SC co‑culture group, 5 × 10^5^ hSCs, or 1 × 10^6^ RSC96 were seeded in a Transwell insert (0.4 μm pore size, Corning, Inc.) the night before scratch assay and the insert was moved onto indicated six-well plate after scratching the cancer cells. Images were captured using an Olympus IX71 digital camera (Center Valley, PA, USA) and analyzed using ImageJ software (NIH). The migration rate was calculated using the following formula: Migration rate = (wound width at 0 h - wound width after incubation) / wound width at 0 h × 100%.

### EdU proliferation assay

Tumor cells (5 × 10^5^; AsPC-1, MIA PaCa-2) were seeded in six-well plates and incubated with complete medium for 12 h. The medium was then replaced with complete medium (control group), complete medium: SCM = 1:1 (SCM group), or co-cultured with 5 × 10^5^ hSCs or 1 × 10^6^ RSC96 using a Transwell insert (0.4 μm pore size, Corning Inc.) in complete medium and cultures were maintained for 24 h. Thereafter, the 5-Ethynyl-2´-deoxyuridine (EdU) proliferation assay was performed with BeyoClick™ EdU-555 detection kits (Beyotime) according to the manufacturer's instructions.

### Enzyme-linked immunosorbent assay (ELISA)

The levels of secreted IL6 and IL1β in cell culture supernatant were determined using a human IL6 Quantikine ELISA Kit and human IL1 beta/IL1F2 Quantikine ELISA Kit (R&D Systems, Minneapolis, MN, USA) according to manufacturer's instructions.

### Small interfering RNA-mediated gene silencing and lentiviral transfection

Scrambled control small interfering RNA (siRNA) or two target-specific siRNA were used to interfere with the expression of corresponding targets using Lipofectamine® RNAiMAX Reagent (Invitrogen, Carlsbad, CA, USA). In brief, cells were seeded in six-well plates and cultured in complete medium before transfection. When the cells reached 50% confluency, we diluted 9 μL of Lipofectamine^®^ RNAiMAX Reagent in 150 μL of Opti-MEM^®^ medium. Then 3 μL of siRNA (10 μM) diluted in 150 μL Opti-MEM^®^ medium was added to the diluted Lipofectamine^®^ RNAiMAX Reagent at 1:1 ratio, and the siRNA-lipid complex was incubated at room temperature for 5 min. Thereafter, the siRNA-lipid complex was added to cells, which were cultured for 48 h. Western blotting or ELISA assays were performed to assess the transfection efficiency. All siRNA oligomers were obtained from Genepharma (Shanghai, China) and their nucleotide sequences are listed in **Table S2**.

For the construction of AsPC-1 cells stably expressing luciferase (luc-AsPC-1), the luciferase expression vector (pLV-CMV-FLuc-PGK-Neo, Jiying Biotech, Shanghai, China) together with vectors psPAX2 and pMD2.G were transduced into HEK-293T cells using Lipofectamine 3000 (Invitrogen) according to manufacturer's instructions, aiming to produce lentiviral particles. Subsequently, AsPC-1 cells were plated in six-well plates and transfected with the packaged lentivirus after reaching approximately 50% confluence. Two days after transfection, cells were screened and purified using neomycin (APExBIO, Houston, TX, USA) for 2 weeks.

### Experimental design of the* in vivo* assay

Twenty severe combined immunodeficiency (SCID) mice aged 4 to 5 weeks were used to perform an* in vivo* metastasis assay in this study. The animal experiment was approved by the South China University Animal Ethics Committee. In brief, 2 × 10^6^ AsPC-1 cells (resuspended in 200 μL FBS-free medium) stably expressing luciferase (luc-AsPC-1) were injected into the lateral tail vein of SCID mice using insulin needles. The mice were randomly divided into four groups, which were injected with (1) non-treated luc-AsPC-1, (2) luc-AsPC-1 co-cultured with hSCs for 48 h, (3) luc-AsPC-1 co-cultured with hSCs in the presence of IL6 neutralizing antibodies (50 ng/mL) for 48 h, (4) luc-AsPC-1 co-cultured with hSCs plus IL1β neutralizing antibodies (40 ng/mL) for 48 h, respectively. Four weeks after injection, the mice in each group were anesthetized using isoflurane inhalation and then injected i.p. with VivoGlo luciferin (150 mg/kg, Promega, Madison, WI, USA). Images were acquired ten minutes after injection using a Bruker In-Vivo Xtreme imaging system (Bruker, Karlsruhe, Germany) and the exposure time was 2 minutes. All mice were then euthanized and their lungs were examined for metastasis via hematoxylin and eosin (H&E) staining.

### Statistical analysis

All results, when appropriate, were described as the mean ± SD. Statistical analysis was performed using SPSS 22.0 (IBM Corp., Armonk, NY, USA). Students' t test (two-tailed) or one-way analysis of variance (ANOVA) was applied for comparisons between two or multiple groups, respectively. The associations between the level of intra-tumoral SC markers in PDAC samples and clinicopathological characteristics were determined using the *χ*^2^ test or Fisher's exact test. Cox proportional hazards models were utilized to determined independent prognostic factors of overall survival on the basis of univariate analysis. Kaplan-Meier survival curves were generated and analyzed using the log-rank test. The Spearman correlation coefficient was determined to analyze the relationship between IL6 and p-NF-kB (p65) immunostaining intensity in PDAC specimens. Statistical significance was accepted at *p* < 0.05.

## Results

### Schwann cells are extensively distributed in the TME and correlate with patients' clinicopathological features and overall survival

**Figure 1:**
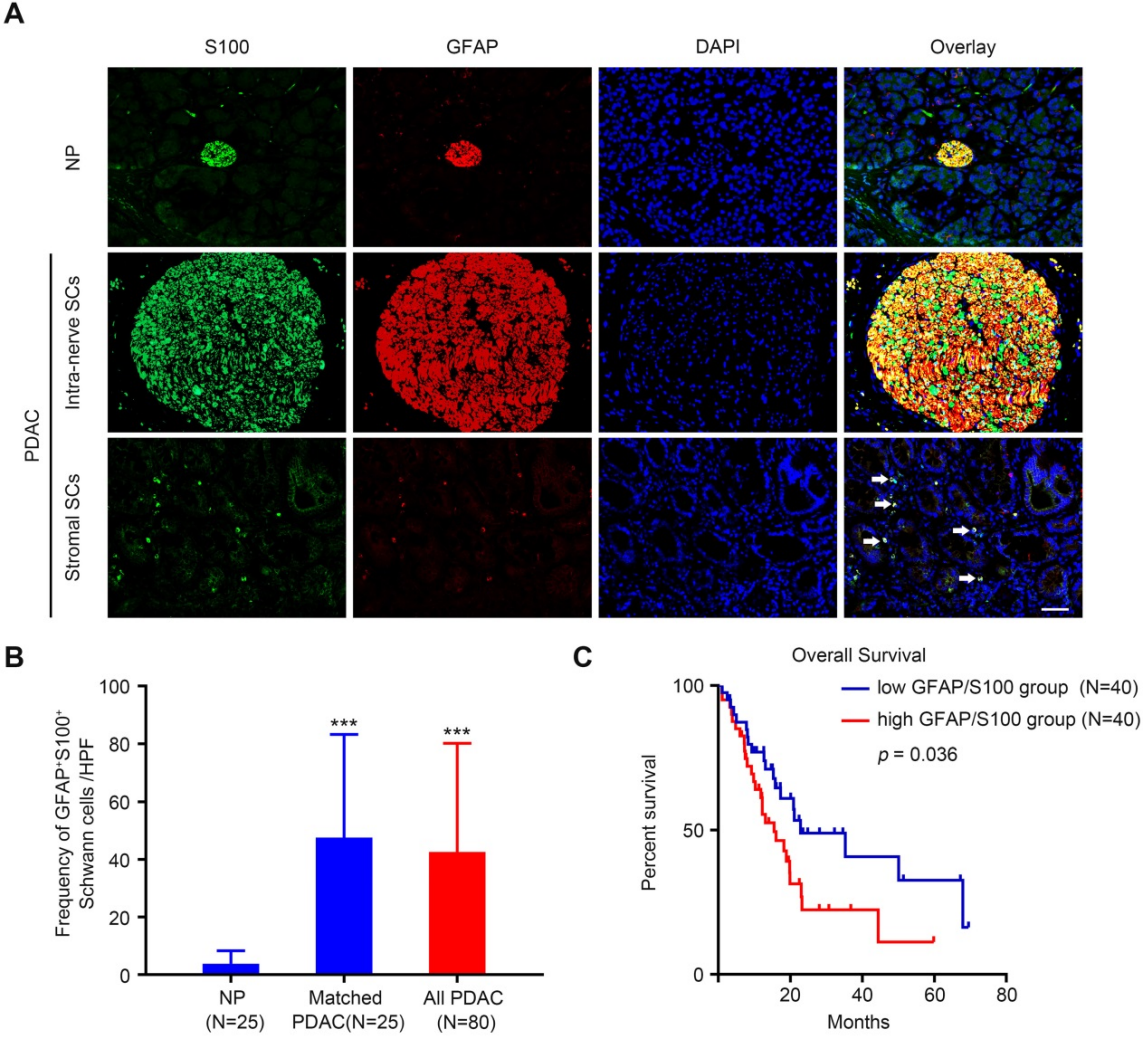
** The distribution of Schwann cells in PDAC and normal pancreas (NP) tissues. A**. Representative double immunolabeling fluorescent images showing that SCs are relatively scarce in NP (**upper panel**), but are widely distributed in the PDAC TME. In PDAC tissues, SCs were detected as present not only within nerves (**middle panel**), but also outside nerves (**lower panel**, **white arrows**). Scale bar: 50 μm. **B**. Compared with NP, PDAC tissues exhibited an elevated SC density. A paired Students' t test or an unpaired Students' t test were applied for the statistical analyses. **C**. Kaplan-Meier survival analysis based on the intra-tumoral SC density of 80 patients with PDAC (log-rank test). *p < 0.05, **p < 0.01, ***p < 0.001.

To identify the presence of SCs in the PDAC stroma, a double immunofluorescence assay was performed using two antibodies against traditional SC markers S100 and glial-fibrillary-acidic protein (GFAP) 11,15. The results showed that SCs were relatively scarce in adjacent normal pancreas tissue (**Figure 1A, upper panel**). By contrast, in the cancerous tissue, SCs were detected not only within nerves (**Figure 1A, middle panel**), but also were scattered in the stroma (**Figure 1A, lower panel**). In some regions, SCs were observed to contact with the epithelial compartment directly (**Figure S1A**). To further confirm the identity of cells detected by these markers, we additionally carried out triple immunofluorescence assay with S100, GFAP, and ALDH1L1, a highly specific astrocyte marker. As shown in **Figure S1B**, immunofluorescent signals of these three markers almost completely overlapped. Quantitative analysis of the frequency of GFAP^+^/S100^+^ SCs detected in the tissue slides demonstrated that SCs were more abundant in the PDAC tumor microenvironment compared with that in the adjacent NP (**Figure 1B**). Kaplan-Meier analysis showed that high level of intra-tumoral SCs markers (GFAP^+^/S100^+^) was associated with a dismal overall survival rate for patients with PDAC (*p* = 0.036,** Figure** 1C). Correlation analysis demonstrated that high level of intra-tumoral GFAP^+^/S100^+^ correlated with a predominantly higher incidence of distant metastases (*p* = 0.046), vascular invasion (*p* = 0.014), and perineural invasion (*p* = 0.025) (**Table 1**). Furthermore, multivariate Cox regression analysis revealed that the level of intra-tumoral GFAP^+^/S100^+^ could serve as an independent prognostic factor for patients with PDAC (*p* = 0.038, **Table 2**).

### Schwann cells augment pancreatic cells migration, invasion, and epithelial‑mesenchymal transition *in vitro*

**Figure 2:**
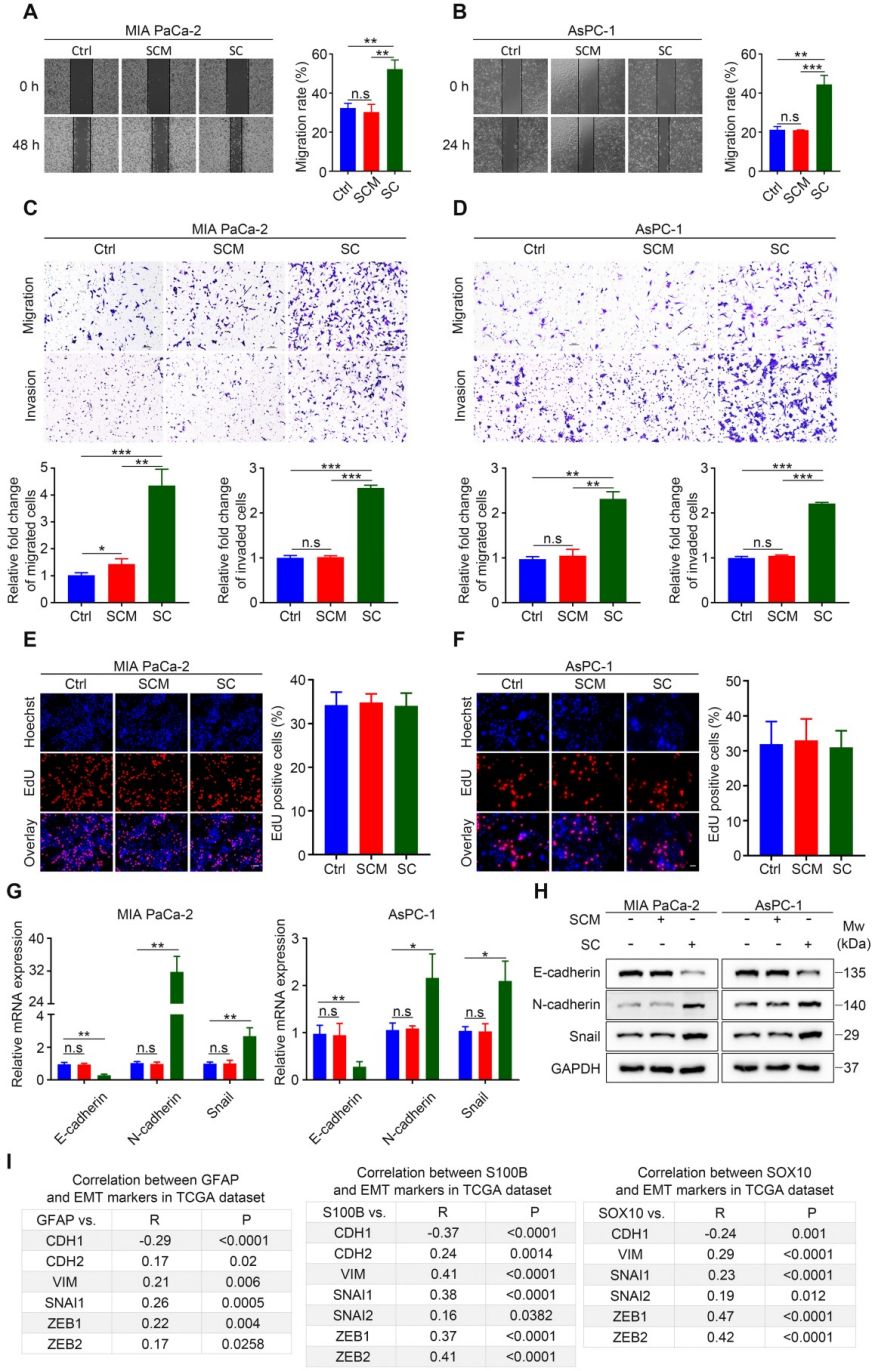
** Schwann cells increase the invasiveness of pancreatic cells and induce EMT *in vitro*. A** and **B**. Representative images of wound healing assays showing that human SCs co-culture enhances the migration of MIA PaCa-2 and AsPC-1 cells, while hSCs conditioned medium (SCM) has no effect on the cell motility of MIA PaCa-2 and AsPC-1 cells. Medium supplemented with 1% FBS was used as a control. **C** and **D**. Representative images of Transwell migration and invasion assays showing that human SCs co-culture enhances the migration and invasion of MIA PaCa-2 and AsPC-1 cells, whereas human SCM has no such effects on MIA PaCa-2 and AsPC-1 cells. Medium containing 1% FBS was used as a control. Scale bar: 100 μm. **E** and **F**. Representative images of EdU proliferation assays showing that neither human SC co-culture nor human SCM affected the proliferation of MIA PaCa-2 and AsPC-1 cells *in vitro*. Scale bar: 50 μm. **G**. QRT-PCR analyses showing that human SC co-culture could downregulate *CDH1* (E-cadherin) and upregulate the expression of* CDH2* (N-cadherin) and EMT regulator *SNAI1* simultaneously, indicating epithelial-mesenchymal transition (EMT) of pancreatic cancer cells after co-cultivation with hSCs. Cancer cells were cultured in control medium (3% FBS/DMEM) or 70% human SCM (SCM: 10% FBS/DMEM = 7:3) or co-cultured with hSCs for 24 h. **H**. Immunoblotting analysis of E-cadherin, N-cadherin and Snail expression in tumor cells in response to human SCM incubation or hSCs co‑culture for 48 h. Cells cultured in control medium were used as a control. Loading control: GAPDH. **I**. Correlations between three Schwann cells markers (*GFAP*, *S10*0, and *SOX10*) and EMT markers (*CDH1*, *CDH2*, *VIM*, *SNAI*1, *SNAI2*, *ZEB*1, *ZEB2*) in PDAC were acquired using the publicly available cBioportal tool (TCGA PanCancer Atlas). *p < 0.05, **p < 0.01, ***p < 0.001.

The mechanism by which SCs promote tumor invasion and metastasis in the context of PDAC remains controversial 10,11. Using wound healing assays, we demonstrated that human SCM did not alter the *in vitro* migration rate of cancer cells, while hSCs co-culture without direct contact resulted in enhanced motility of malignant cells (**Figure 2A-B**). Similarly, Transwell migration and invasion assays showed that cancer cells co-cultured with human SCs exhibited a more migratory and invasive phenotype compared with their non-treated or SCM-treated counterparts (**Figure 2C-D**). Interestingly, human SCM slightly increased the migration rate of MIA PaCa-2 cells. Moreover, enhanced migration and invasion were also observed in MIA PaCa-2 and AsPC-1 cells co-cultured with RSC96 cells compared with cancer cells in the control group or those treated with RSC96 derived SCM (**Figure S2A-D**). These results indicated that both human and rat SCs co‑cultured with cancer cells acquired a tumor-promoting phenotype compared with non‑educated (co‑cultured) SCs. Besides, EdU incorporation assays demonstrated that both hSCs and RSC96 cells had no impact on MIA PaCa-2 and AsPC-1 cells proliferation (**Figure 2E-F, Figure S2E-F**).

Schwann cells could induce EMT of cancer cells in salivary adenoid cystic carcinoma and lung cancer 8,9, which is important for metastasis in many malignancies. In the present study, qRT-PCR and western blotting analysis confirmed that both human and rat SCs induced EMT of MIA PaCa-2 and AsPC-1 cells, as evidenced by downregulation of E-cadherin and upregulation of N-cadherin and Snail family transcriptional repressor 1 (Snail) (**Figure 2G-H, Figure S2G-H**). Furthermore, by analyzing the publicly accessible datasets for patients with pancreatic adenocarcinoma (PAAD), we found that the gene expression of three typical SC markers, *GFAP*,* S100B*, and SRY-box transcription factor 10 (*SOX10*), was significantly associated with that of several EMT markers (**Figure 2I, Figure S2I**). Taken together, our results showed that SCs acquired the ability to promote pancreatic cancer cell migration, invasion, and EMT after contact-independent co-culture with pancreatic cancer cells, without affecting their proliferation* in vitro*.

### Schwann cell-derived IL6 is responsible for enhanced migration, invasion, and EMT of pancreatic cancer cells

**Figure 3:**
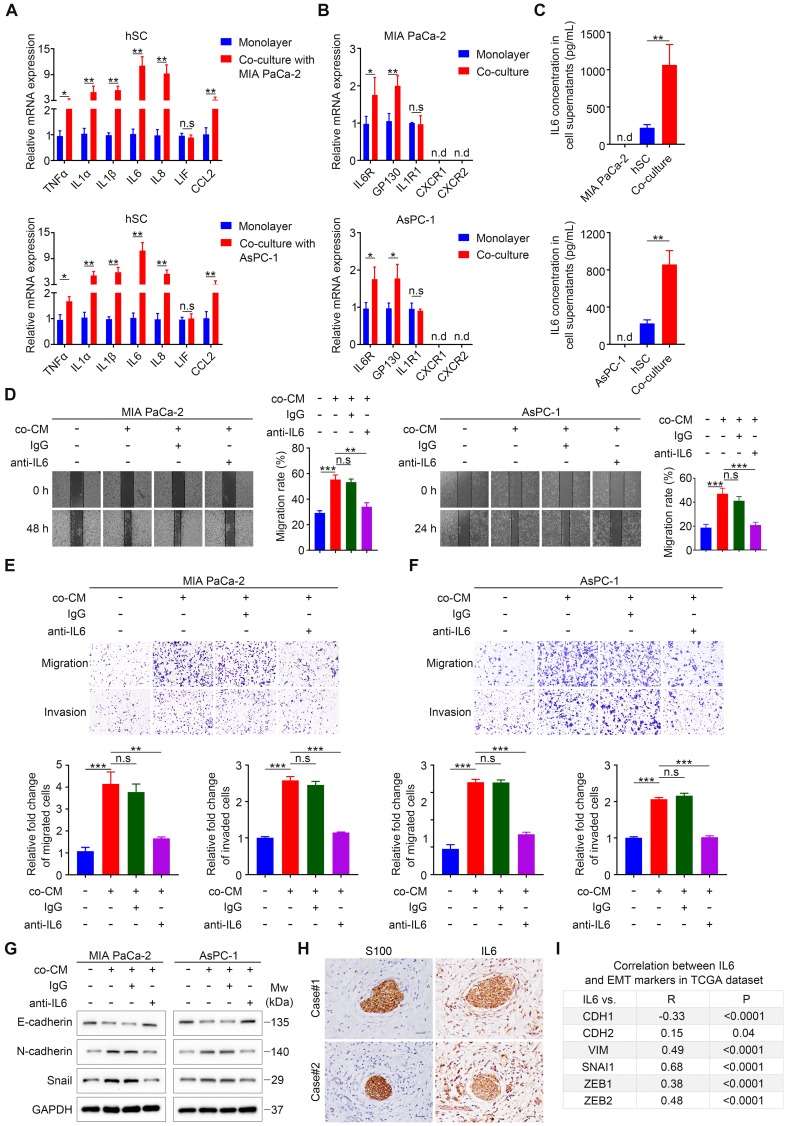
** Schwann cells secret IL6 to promote pancreatic cell migration, invasion, and epithelial-mesenchymal transition *in vitro*. A**. QRT-PCR analysis of inflammatory cytokine mRNAs (*TNFA*, *IL1A*, *IL1B*, *IL6*, *IL8*, *LIF*, *CCL2*) in human SCs cultured in monolayers or co‑cultured with pancreatic cancer cells for 24 h. **B**. The expression of corresponding receptors in pancreatic cancer cells (*IL6R*, *GP130*, *IL1R1*, *CXCR1*, and *CXCR2*) cultured in monolayers or co‑cultured with human SCs was assessed by qRT-PCR. **C**. The protein levels of IL6 in cell supernatants were determined via an ELISA assay. **D**. Representative images of wound healing assay showing that the addition of IL6 neutralizing antibodies (50 ng/mL), rather than isotype control antibodies (50 ng/mL), to co-cultured CM (co-CM) impaired the co-CM-induced pancreatic cells migration. Medium supplemented with 1% FBS was used as a control. **E** and **F**. Representative images of Transwell migration and invasion assays showing that the addition of IL6 neutralizing antibodies (50 ng/mL), rather than isotype control antibodies (50 ng/mL), to co-CM abrogated the co‑CM-induced pancreatic cancer cell migration and invasion. Medium containing 1% FBS was used as a control. Scale bar: 100 μm. **G**. Cancer cells were cultured in control medium (3% FBS/DMEM) or 70% co-CM (co-CM:10% FBS/DMEM = 7:3) in the presence of isotype control antibodies (50 ng/mL) or 50 ng/mL IL6 neutralizing antibodies for 48 h and then collected for to determine the protein levels off E-cadherin, N-cadherin, and Snail using immunoblotting analysis. GAPDH was used as loading control. **H**. Representative images of IHC staining of IL6 and S100 in sequential tissues from two PDAC patients. Scale bar: 20 µm. **I**. Correlations between the gene expression of* IL6* and EMT markers (*CDH1*, *CDH2*, *VIM*, *SNAI1*, *ZEB1*, *ZEB2*) in PDAC were obtained using the publicly accessible cBioportal tool (TCGA PanCancer Atlas). *p < 0.05, **p < 0.01, *** *p*< 0.001.

Our results implied a significant role of paracrine factors in increasing cancer cell motility and invasiveness. To identify these effective factors, we used a co-culture Transwell system (**Figure S3H**), which only allows paracrine tumor-neuroglia interactions, similar to the way by which PCa cells interact with SCs in our aforementioned functional experiments. Increasing studies suggested that SCs undergo phenotypic and functional reprogramming upon exposure to tumor cells 11,13, similar to the reversible dedifferentiation of SCs during nerve injury (termed as repair SCs or immature SCs). Repair SCs are characterized by upregulation of several neurotrophic proteins, such as nerve growth factor (NGF), brain derived neurotrophic factor (BDNF), glial cell derived neurotrophic factor (GDNF), and L1 cell adhesion molecule (L1CAM) 16, all of which have been reported to promote metastasis or PNI in various malignancies 17. However, our qRT-PCR analysis revealed that tumor-neuroglia cell co-cultivation did not significantly alter the expression of these four proteins in SCs and their corresponding receptors in MIA PaCa-2 and AsPC-1 cells at the mRNA level (**Figure S3A-B**). Another hallmark of immature SCs is the activation of an innate immune response, comprising the upregulation of cytokines such as TNFα, IL1α, IL1β, LIF interleukin 6 family cytokine (LIF), and monocyte chemotactic protein 1 (MCP-1) 16. In addition, SCs were reported to secret a dramatically higher levels of IL6 and IL8 under hypoxia 18, all of which have been reported to promote cancer cell metastasis 19,20. Strikingly, SCs co-cultured with MIA PaCa-2 or AsPC-1 cells exhibited elevated levels of pro-inflammatory genes, among which *IL1A* (IL1α),* IL1B* (IL1β),* IL6*, and *IL8* were the most highly upregulated when compared with SCs cultured in monolayers (**Figure 3A**). Furthermore, we found that cancer cells co-cultured with SCs expressed a higher level of the IL6 receptor (IL6R) and the IL6 signal transducer (gp130), respectively. By contrast, we observed no significant change with respect to the mRNA levels of *IL1R1*, which encodes the receptor for IL1α and IL1β, while the expression of C-X-C motif chemokine receptor 1 (CXCR1) and CXCR2, receptors for IL8, were not detectable in MIA PaCa-2 and AsPC-1 cells (**Figure 3B**). Moreover, ELISA assays confirmed that neither MIA PaCa-2 nor AsPC-1 cells alone produced a detectable level of IL6, and SCs alone produced low levels of IL6, while tumor-neuroglia co-cultures showed a four-fold increase of IL6 (**Figure 3C**). Interestingly, neither MIA PaCa-2 nor AsPC-1 cells cultured alone or co-cultured with SCs expressed detectable levels of *IL6* mRNA (**Figure S3C**), indicating that SCs educated by pancreatic cancer cells are the only source of IL6 in co-culture. As a result, we considered IL6 as the most likely paracrine factor that contributes to the enhanced cancer cell migration and invasion. To test this hypothesis, we added neutralizing anti-human IL6 antibodies to the co-cultured conditioned medium and found that although the co-cultured CM dramatically promoted cancer cell migration, invasion, and EMT, the anti-IL6 antibodies significantly abolished these effects (**Figure 3D-G**). To further confirm these results, we performed immunochemistry and immunofluorescence staining of IL6 on two human PDAC tissue sections. Consistently, we observed intensive staining of IL6 in S100-positive SCs via IHC (**Figure 3H**) and co-localization of IL6 and S100 by IF (**Figure S3D**). Furthermore, analysis of publicly available The Cancer Genome Atlas (TCGA) datasets revealed a negative association between IL6 and E-cadherin, a marker for the epithelial phenotype. By contrast, the mRNA level of *IL6* expression positively correlated with that of several markers for the mesenchymal phenotype (**Figure 3I, Figure S3F, left chart**) and SCs traditional markers (**Figure S3E, Figure S3F, right chart**). In addition, a high IL6 level predicted a dismal overall survival for patients suffering from PAAD (**Figure S3G**). Together, these results suggested an important role for SCs-derived IL6 in promoting pancreatic cancer cell migration, invasion, and EMT.

### STAT3 signaling is required for SC-induced pancreatic cancer cells migration and invasion

**Figure 4:**
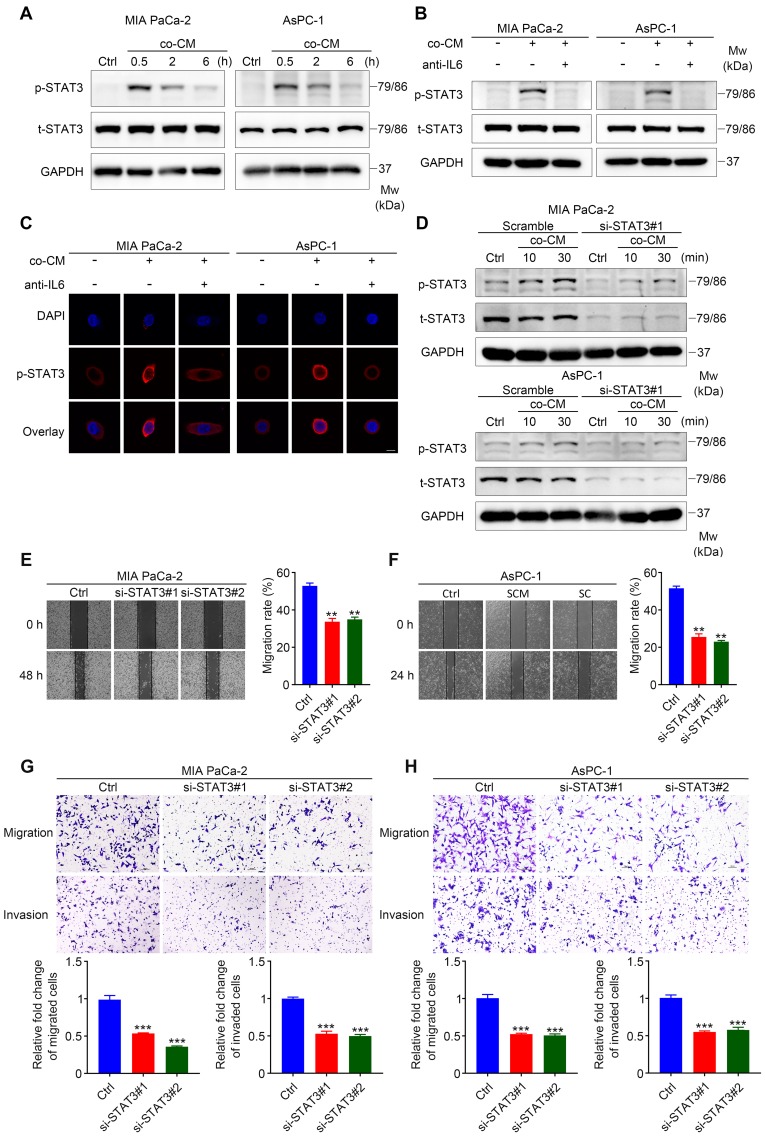
** STAT3 signaling is critical for SC-induced pancreatic cancer cell migration and invasion. A**. Immunoblotting assay demonstrating increased STAT3 phosphorylation in pancreatic cancer cells following exposure to co-cultured conditioned medium (co-CM) for the indicated times. Cells without co-CM treatment were used as a control (Ctrl). **B**. Western blotting of pancreatic cancer cells shows reduced level of phosphorylated STAT3 (p-STAT3) following the addition of IL6 neutralizing antibodies (50 ng/mL) to co-CM compared with those only treated with co-CM. The duration of co-CM treatment in both groups was 30 min. Cells with no treatment were used as a control. **C**. Representative images of the immunofluorescence assay results for anti-p-STAT3 (red) and DAPI (blue) staining. Pancreatic cancer cells were treated with co-CM or co-CM plus IL6 neutralizing antibodies (50 ng/mL) for 30 min and then collected for immunofluorescence analysis. Cells without any treatment were used as a control. Scale bar: 10 μm. **D**. Immunoblotting analysis showing disrupted STAT3 activation following co-CM incubation at the indicated times in *STAT3* siRNA#1 targeted pancreatic cancer cells. Scrambled siRNA targeted cancer cells exposed to co‑CM were used as a control. **E** and **F**. Representative images of wound healing assay showing that *STAT3* interference undermined co-CM induced pancreatic cancer cell migration. Control siRNA targeted tumor cells incubated with co-CM were used as a control. **G** and **H**. Scrambled siRNA and *STAT3* siRNA- targeted MIA PaCa-2 and AsPC-1 cells were used for Transwell migration and invasion assays. Co-CM was used as a chemoattractant in all groups. Scale bar: 100 μm. *p < 0.05, **p < 0.01, ***p < 0.001.

The IL6 signaling cascade begins with the binding of IL6 to IL6R, followed by the association of IL6R and gp130 21, which induces the phosphorylation of STAT3 and the formation of STAT3 homodimers, followed by the translocation of STAT3 homodimers into the nucleus to modulate the expression of IL6-responsive genes 22. To identify whether STAT3 signaling is required for SC-mediated pancreatic cancer cell migration and invasion, we first determined whether the STAT3 pathway was activated in MIA PaCa-2 and AsPC-1 cells treated with co-cultured CM. Western blotting assays showed that the addition of co-cultured CM to cancer cells led to dramatic STAT3 activation (**Figure 4A**). Consistently, immunofluorescence assays confirmed STAT3 activation in MIA PaCa-2 and AsPC-1 cells exposed to co-cultured CM, as shown by enhanced phospho-STAT3 fluorescent signal in the cytoplasm and nuclear translocation (**Figure 4C**). Importantly, these effects were inhibited when neutralizing antibodies against IL6 were added (**Figure 4B, 4C**). All these results suggested that STAT3 signaling was activated by paracrine IL6 secreted by educated SCs.

Next, we determined whether SC-induced IL6 signaling functioned through gp130 expressed on the membrane of cancer cells. Interfering with gp130 expression effectively inhibited STAT3 phosphorylation in tumor cells exposed to co-cultured CM (**Figure S4A-B**), and partially impaired the co-cultured CM-induced migration, invasion, and EMT of pancreatic cancer cells (**Figure S4C-E**). In addition, we proved that SC-induced IL6 signaling did not operate via IL6 trans-signaling using sgp130Fc (**Figure S4F**), which selectively inhibits the trans-signaling via targeting the IL6-sIL6R complex 23. To further determine whether STAT3 signaling is indispensable for enhanced cancer cell motility and invasiveness, two siRNAs against *STAT3* and a control (scrambled) siRNA were used to disrupt *STAT3* expression in MIA PaCa-2 and AsPC-1 cells. Notably, although co‑cultured CM significantly increased the level of phospho-STAT3 in MIA PaCa-2 and AsPC-1 cells targeted by the scrambled siRNA, cancer cells targeted by two *STAT3*-specific siRNAs exhibited impaired phospho-STAT3 induction following co-cultured CM treatment (**Figure 4D**, **Figure S4G**). Using would healing assays, Transwell assay, and immunoblotting analysis, we demonstrated that STAT3 downregulation significantly impaired co-cultured CM-induced pancreatic cancer cell migration, invasion, and EMT (**Figure 4E-H, Figure S4H**). Taken together, these results suggested a vital role for STAT3 signaling in SC-derived IL6-induced pancreatic cancer cell migration and invasion.

### Activated NF-κB Signaling is required for upregulated IL6 production from SCs

**Figure 5:**
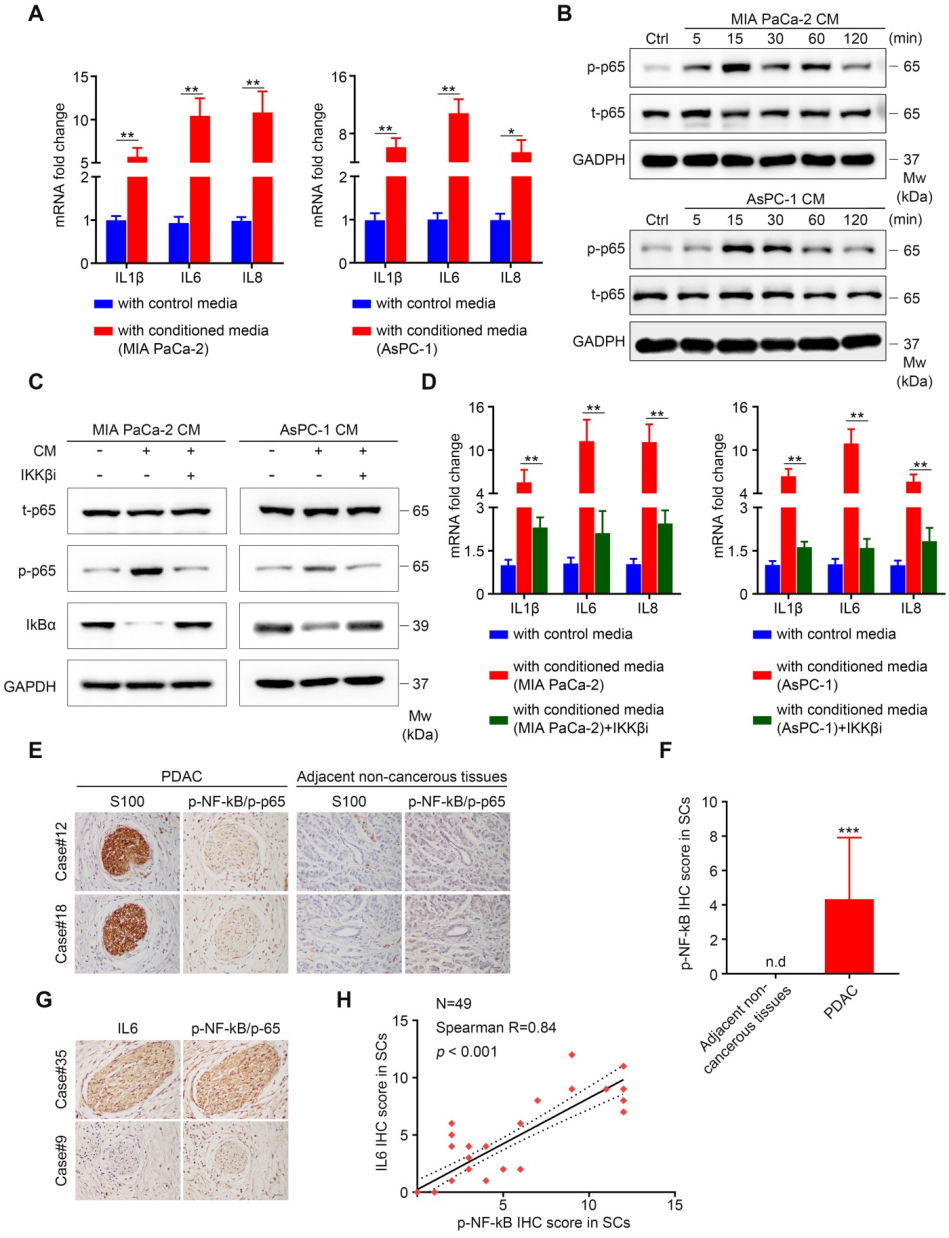
** Active NF-κB signaling is responsible for elevated IL6 production from SCs. A**. Human SCs were cultured in control medium (3% FBS/DMEM) or 70% TCM (TCM: 10% FBS/DMEM = 7:3) for 24 h and subsequently collected for qRT-PCR analysis. **B**. Human SCs were treated with TCM at the indicated times and then collected for immunoblotting analysis of phosphorylated p65 (p-p65) and total p65 (t-p65). Cells without TCM exposure were used as a control. Loading control: GAPDH. **C**. Western blotting analysis of p-p65, t-p65 and total IkBα in human SCs cultured in control medium or TCM in the absence or presence of 40 μmol/L IKKβ inhibitor (IKKβi) ML120B for 15 min. Loading control, GAPDH. **D**. QRT-PCR analysis of *IL1B*, *IL6*, and *IL8* mRNA expression in human SCs cultured in control medium or 70% TCM in the absence or presence of 40 μmol/L IKKβ inhibitor (IKKβi) ML120B for 24 h. **E**. Representative IHC images showing intensive p-NF-κB/p-65 staining in S100-positive SCs in surgically resected PDAC samples, but not in SCs in the adjacent non-cancerous tissues from two patients. Scale bar: 20 μm. **F**. Differential analysis of p-NF-κB/p-65 IHC score between PDAC samples (N = 49) and adjacent non-cancerous tissues (N = 25). An unpaired Students' t test was applied for statistical analysis. **G**. Representative IHC images showing substantial overlap of IL6 and p-NF-κB/p-65 staining in PDAC tissues from two patients. Scale bar: 20 μm. **H**. Correlation analysis of IL6 and p-NF-κB/p-65 IHC score in SCs (N = 49). *p < 0.05, **p < 0.01, ***p < 0.001.

Next, we started to determine the critical intracellular mediators responsible for increased production of IL6 from SCs. Notably, our results showed that tumor-neuroglia co-culture increased IL1β, IL6, and IL8 expression in SCs, all of which are well-known targets of nuclear NF-κB 24. Consequently, we hypothesized that NF-κB signaling might be responsible for the upregulated expression of pro‑inflammatory genes in SCs. To start with, we confirmed that conditioned medium from MIA PaCa-2 and AsPC-1 cells was sufficient to enhance IL1β, IL6, and IL8 expression in SCs (**Figure 5A**). Next, we investigated whether NF-κB pathway in SCs was activated following tumor cells conditioned media (TCM) treatment. Western blotting results confirmed that activation of p65 occurred rapidly in SCs upon the addition of TCM, as shown by the increased level of phospho-p65, parallel to phosphorylation of IKK and IκBα, which was followed by its degradation (**Figure 5B, Figure S5A-B**). Having confirmed the activation of NF-κB pathway after TCM exposure, we subsequently utilized ML120B, an IKKβ inhibitor (IKKβi), to investigate whether NF-κB pathway activation was required for upregulated IL1β, IL6, and IL8 expression in SCs. Our results demonstrated that the pre-addition of ML120B into culture medium of SCs impaired NF-κB pathway activation, as shown by decreased levels of phospho-p65 and stabilization of total IκBα (**Figure 5C**). Furthermore, we observed a significant decrease in *IL1B*, *IL6*, and* IL8* mRNA levels in SCs treated with TCM in the presence of IKKβi compared with those only treated with TCM (**Figure 5D**). To further confirm these results, we performed immunochemical staining of IL6 and p-NF-κB/p-p65 in PDAC tissue samples and revealed that the staining intensity of p-NF-κB/p-65 (S536) in S100 positive SCs was more intensive in the PDAC microenvironment compared with that in the adjacent non-cancerous tissues (**Figure 5E-F**). Moreover, we observed a strong correlation between the staining intensity of IL6 and p-NF-κB/p-65 in SCs (**Figure 5G-H**). Taken together, these results demonstrated that the NF-κB pathway is involved in the elevated cytokine expression in tumor‑conditioned SCs.

### PDAC cell-secreted IL1β is responsible for NF-κB pathway activation and increased expression of pro-inflammatory cytokines in SCs

**Figure 6:**
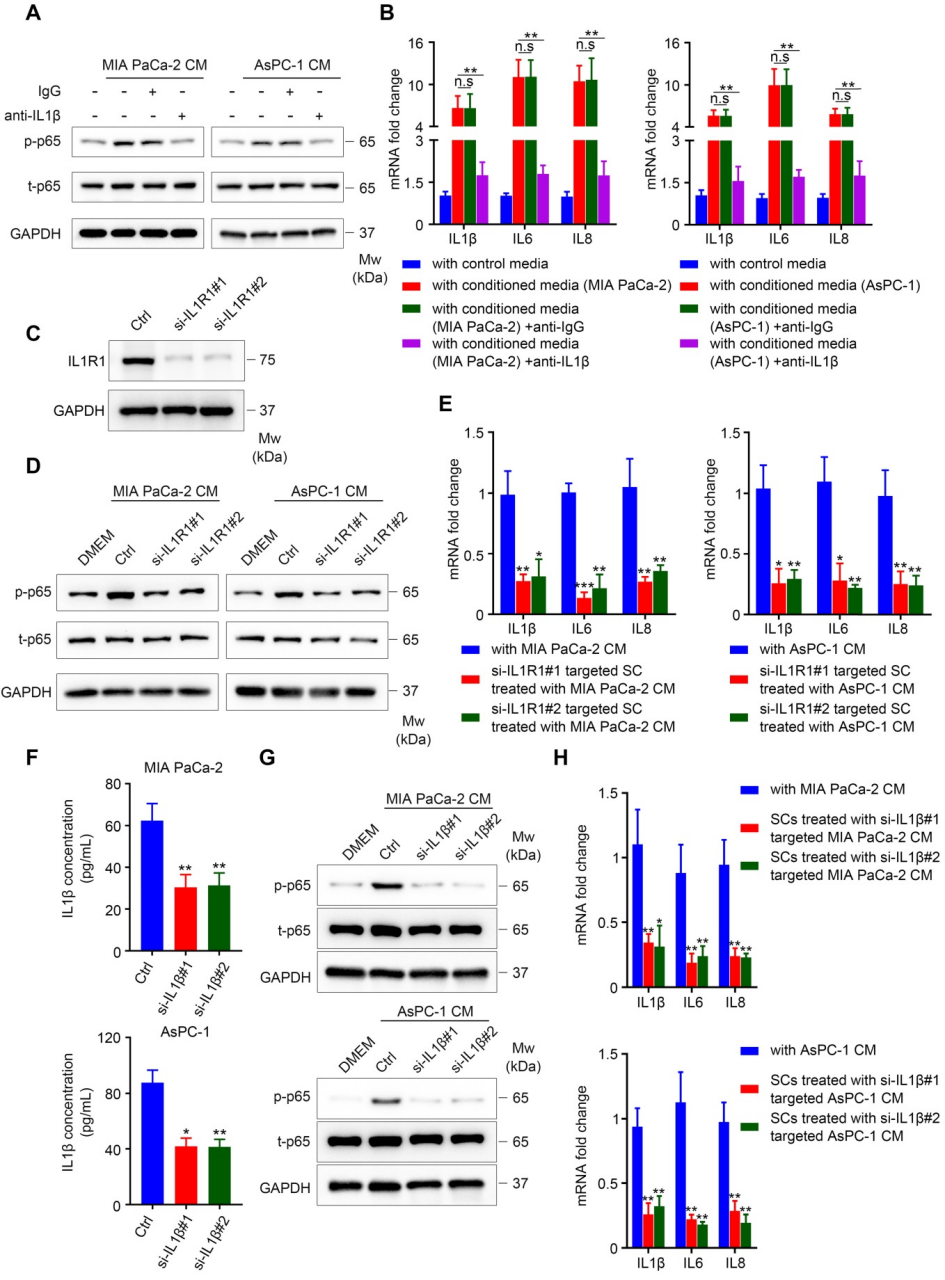
** IL1β derived from pancreatic cells activates the NF-κB pathway in SCs and upregulates pro-inflammatory cytokine production from SCs. A**. Immunoblotting analysis of p‑p65 and t-p65 in human SCs cultured in control medium or TCM in the absence or presence of 40 ng/mL isotype control antibodies or IL1β neutralizing antibodies for 15 min. Loading control, GAPDH. **B**. QRT‑PCR analysis of *IL1B*, *IL6*, and *IL8* mRNA levels in human SCs cultured in control medium or 70% TCM in the absence or presence of 40 ng/mL isotype control antibodies or IL1β neutralizing antibodies for 24 h. **C**. Western blotting was performed to confirm the knockdown efficiency of *IL1R1* in human SCs treated with two* IL1R*1-targeted siRNAs. Loading control, GAPDH. **D**. Immunoblotting analysis of p-p65 and t-p65 in human SCs showing that knockdown of *IL1R1* in SCs impaired NF-κB (p65) pathway activation after TCM treatment. GAPDH was used as the loading control. **E**. QRT-PCR analysis of* IL1B*, *IL6*, and *IL8* mRNA levels in scrambled siRNA or *IL1R1*-specific siRNA targeted human SCs cultivated with 70% TCM for 24 h. **F**. siRNA mediated *IL1B* knockdown in MIA PaCa-2 and AsPC-1 cells was verified using ELISA. **G**. Immunoblotting analysis of p-p65 and t-p65 in human SCs exposed to control medium, control TCM, or TCM derived from *IL1B* knockdown pancreatic cancer cells for 15 min. Loading control, GAPDH. **H**. QRT-PCR analysis of *IL1B*, *IL6*, and *IL8* mRNA levels in human SCs cultured in control 70% TCM or 70% TCM derived from *IL1B* knockdown pancreatic cancer cells for 24 h. *p < 0.05, **p < 0.01, ***p < 0.001.

Our results showed that TCM activated the NF-κB/p-65 pathway in SCs, which was accompanied by elevated levels of phospho-IKK and IκBα, and followed by IκBα degradation, suggesting the activation of the canonical NF-κB pathway (**Figure S5A-B**) 25. The classical pathway is activated by proinflammatory cytokines (TNFα and IL1), pathogen associated molecular patterns (PAMPs) and damage-associated molecular patterns (DAMPs) 26. Here, we chose TNFα and IL1β as candidate factors responsible for NF-κB pathway activation in SCs exposed to TCM for the following reasons: (1) Human SCs co-cultured with pancreatic cancer cells exhibited elevated expression of several pro-inflammatory cytokines and chemokines, indicating an immature phenotype; (2) immature SCs are characterized by re-expression of molecules that were suppressed when immature SCs started to myelinate during development, including GFAP 16; and (3) pro‑inflammatory cytokines (IL1β, TNFα, and lipopolysaccharide (LPS)) were reported to induce GFAP expression in enteric glia 27. Both TNFα and IL1β were sufficient to induce the expression of pro-inflammatory genes (**Figure S6A-B**). Nevertheless, neutralizing TNFα was not sufficient to impair the induction of pro-inflammatory cytokines in TCM-conditioned SCs (**Figure S6C-D**). By contrast, the addition of anti-IL1β neutralizing antibodies into TCM significantly inhibited the ability of MIA PaCa-2 or AsPC-1 CM to phosphorylate NF-κB/p65 (**Figure 6A**) and effectively impaired the induction of IL1β, IL6 and IL8 in SCs (**Figure 6B**). Furthermore, when treated with MIA PaCa-2 or AsPC-1 conditioned medium, SCs targeted by two specific siRNAs against *IL1R1* (**Figure 6C**) exhibited reduced phosphorylation of p-NF-κB/p-65 (**Figure 6D**) and downregulated expression of *IL1B*, *IL6*, and *IL8* (**Figure 6E**) compared with those in scrambled siRNA-targeted SCs. To further confirm the role of the IL1β-IL1R1 axis in activating the NF-κB pathway and inducing pro-inflammatory cytokine expression in TCM-treated SCs, we utilized two siRNAs against *IL1B* to knockdown IL1β levels in tumor cells, which was validated using ELISA (**Figure 6F**). As expected, the phosphorylation of NF-κB/p-65 and the expression of *IL1B*, *IL6*, and *IL8* were significantly inhibited in SCs treated with conditioned medium derived from *IL1B*-knockdown MIA PaCa-2 or AsPC-1 cells compared with cells targeted by the scrambled siRNA (**Figure 6G-H**). Taken together, these results showed that IL1β secreted from pancreatic cancer cells could activate NF-κB signaling in SCs via IL1R1 to promote pro-inflammatory cytokine production.

### Disrupting the tumor-neuroglia interaction impaired tumor metastasis *in vivo*

**Figure 7:**
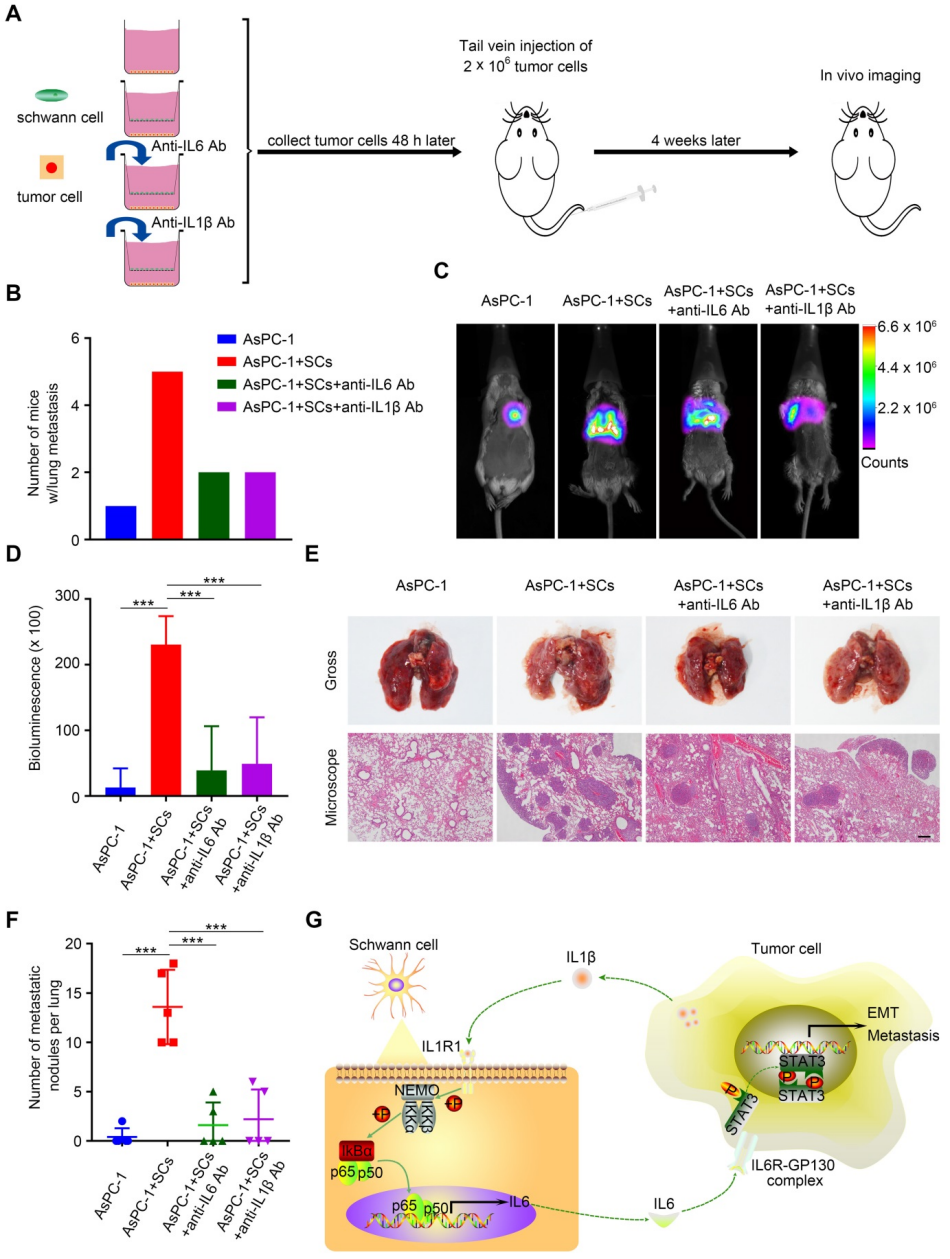
** The tumor-neuroglia interaction facilitates cancer dissemination *in vivo*. A**. Schematic illustration of the animal experiment design. In brief, luc-AsPC-1 cells were cultured alone or co-cultivated with human SCs, or co-cultured with human SCs in the presence of IL6 or IL1β neutralizing antibodies. Forty-eight hours later, luc-AsPC-1 cells were digested and injected into the lateral tail vein of SCID mice. Four weeks after injection, lung metastasis was detected using an* in vivo* imaging system. **B**. Numbers of mice that developed lung metastasis in each group at the end of experiment are shown. **C**. Representative images of mice that developed lung metastasis in each group are shown, as visualized using the Bruker In-Vivo Xtreme imaging system. **D**. Quantitative analysis of the bioluminescence intensity from the lung were acquired using the Bruker molecular imaging software. **E**. At the end of animal experiment, all mice were sacrificed and their lungs were excised, photographed, and sectioned. The upper panel presents the macroscopic appearance of metastatic lung tumors; the lower panel presents the H&E staining. Scale bar:100 μm. **F**. Statistical analysis of metastatic nodules in the lung. **G**. Schematic illustration of the crosstalk between SCs and pancreatic cancer cells. Tumor cells secrete IL1β to activate the NF-κB pathway in SCs and increase IL6 production from SCs, which binds to IL6R on cancer cells and promotes PCa cell metastasis via the activation of STAT3 signaling. In summary, SCs could acquire a tumor-promoting phenotype by interacting with pancreatic cancer cells.

Next, we investigated the impact of the tumor-neuroglia interaction on pancreatic cancer cells metastasis in an *in vivo* animal model. Forty-eight hours after *in vitro* incubation, luc-AsPC-1 cells were digested and injected into the lateral tail vein of SCID mice. Four weeks after injection, lung metastasis was detected using an *in vivo* imaging system (**Figure 7A**). Strikingly, all mice injected with luc-AsPC-1 cells co-cultured with human SCs developed lung metastasis, while only one out of five mice exhibited lung metastasis in the monolayer culture group (**Figure 7B**). Furthermore, the addition of IL6 or IL1β neutralizing antibodies to the co-culture system between luc-AsPC-1 cells and human SCs significantly impaired cancer cell dissemination *in vivo*, as shown by a lower incidence of lung metastasis (**Figure 7B**) and a decreased bioluminescence signal (**Figure 7C-D**). Moreover, H&E staining confirmed that pancreatic cancer cells co-cultured with human SCs formed more and larger metastatic nodules in the lung compared with those cultured as a monolayer. Notably, adding anti-IL6 or anti-IL1β neutralizing antibodies to the co-culture system for the purpose of interrupting the tumor-neuroglia interaction significantly inhibited the development of lung metastasis (**Figure 7E-F**). These results suggested that targeting the tumor-neuroglia crosstalk impeded pancreatic cancer cell dissemination *in vivo.*

## Discussion

The tumor microenvironment is critical for pancreatic cancer progression. Pancreatic nerves, one component of the peripheral nervous system (PNS) in the PDAC TME, undergo prominent alterations during the evolution and development of pancreatic cancer 28. These neuropathic alterations, consisting of neural hypertrophy (increased nerve size), increased nerve numbers, nerve remodeling (altered proportion between sensory and autonomic fibers), neuritis and perineural invasion, have prognostic value for patients with PDAC. Recently, the peripheral nervous system has attracted increasing interest for its role in tumor initiation and progression 29-32. For example, autonomic nerve fibers formation contributed to prostate cancer initiation and dissemination in a transgenic mouse model 29. Zahalka demonstrated that adrenergic nerve-derived noradrenaline binds to the β‑adrenergic receptor on endothelial cells to induce angiogenesis, thus accelerating aberrant tumor growth in prostate cancer 30. In PDAC, depletion of sensory neurons by neonatal capsaicin treatment delayed tumor formation and progression in a genetically engineered mouse model (GEM) 31.

Until recently, SCs, another component of the PNS, were underestimated for their roles in tumor development, apart from nervous system neoplasms. Schwann cells, which are the main neuroglia with high plasticity in the PNS, have attracted extensive attention recently. Accumulating evidence demonstrates that SCs have an active role in tumor progression. For example, Sroka showed that the myelinating phenotype of SCs promoted integrin-dependent tumor invasion in prostate and pancreatic cancer 33. Shurin reported that SCs augment cancer cells metastasis in lung cancer 8 and possess an immunomodulatory capability in shaping the TME in melanoma, thereby aiding tumor growth 13. However, the role of SCs in tumor initiation and progression have been largely limited to perineural invasion (PNI) in PDAC 10,11, which is an important independent prognostic factor in a variety of malignancies. In the present study, we demonstrated that, in contrast with adjacent normal pancreas, SCs are widely distributed in the PDAC TME, and could be divided into intra-nerve SCs and stromal SCs. More importantly, to the best of our knowledge, this was the first demonstration that a high level of intra-tumoral SCs markers could serve as an independent prognostic factor for poor survival of patients with PDAC and correlated with a higher incidence of distant metastases, vascular invasion, and PNI.

Cells from the TME might contribute to tumor metastasis via paracrine signaling, direct contact with tumor cells, or by matrix remodeling 11, including SCs 10,11. SCs are reported to promote cancer cell protrusion formation and detachment of individual cancer cell from cancer cells clusters in a contact-dependent manner, thereby aiding cancer invasion and PNI 11. Besides, SCs facilitate PNI through paracrine L1CAM 10. In that study, L1CAM promoted neural dissemination via two mechanisms: (1) By functioning as a chemoattractant for cancer cells; and (2) facilitating matrix remodeling along the axon through inducing matrix metalloproteinase production from cancer cells via STAT3 signaling activation. However, the interactions between tumor cells and SCs through paracrine signaling, and their roles in tumor metastasis, remain unclear. In the present study, we demonstrated that both human SC and rat SC co-culture could increase pancreatic cancer cells motility and invasiveness in an *in vitro* Transwell system that prevented SCs and tumor cells from directly contacting with each other. By contrast, neither human SCM nor rat SCM were sufficient to facilitate pancreatic cancer cell migration and invasion, implying an important role of intercellular communication between tumor cells and SCs in endowing SCs with a tumor-promoting phenotype. Consistently, our subsequent experiments proved that tumor cells secret IL1β to activate the NF-κB pathway in SCs. Reciprocally, educated SCs secret IL6 to activate STAT3 signaling in cancer cells, thus augmenting cancer cell invasion and metastasis *in vitro* and *in vivo*.

IL6, a typical pleiotropic cytokine, has been proven to accelerate tumorigenesis and tumor progression in various cancers, either through direct effects on cancer cells (for instance, cell survival, proliferation, and metastasis) or via its extrinsic effects on the TME, such as modulation of tumor-infiltrating immune cells, which creates an immunosuppressive TME favorable for tumor growth and metastasis 23. IL6 is produced by tumor cells themselves and diverse cell types in the TME, such as cancer-associated fibroblasts (CAFs) and tumor-associated macrophages (TAMs), both of which have been reported to promote cancer stem cell properties through paracrine IL6 34,35. Interestingly, Demir observed a significantly higher level of IL6 production from human SCs under hypoxia 18, a prominent feature of the PDAC TME, indicating that SCs might be an alternative source of IL6. Consistently, our IHC results confirmed the extensive presence of IL6 in S100-positive SCs. Nevertheless, to date, the role of SC-secreted IL6 in the tumorigenesis and development of PDAC remains unknown. In the present study, we showed that SCs are the only source of IL6 in our co-culture system. Furthermore, SC-derived IL6 induced pancreatic cancer cells migration and invasion via activating STAT3 signaling *in vitro*. Meanwhile, all of these effects were abrogated either by adding IL6 neutralizing antibodies to the co‑cultured CM or by downregulating STAT3 or gp130 expression in cancer cells. In a metastatic mouse model, compared with non-treated tumor cells, cancer cells co-cultured with SCs before injection exhibited a higher metastatic incidence and formed more metastatic nodules in the lung. By contrast, the addition of IL6 neutralizing antibodies to the co-culture system significantly inhibited tumor dissemination. These results emphasized the role of IL6 originating from educated SCs in facilitating tumor dissemination.

The NF-κB pathway, which is constitutively activated in multiple cancers, plays an important role in tumor initiation, promotion, and progression, including PDAC 25. The NF-κB pathway has been well studied for its roles in SCs differentiation. In rat sciatic nerves, NF-κB was reported to be significantly upregulated in pre-myelinating SCs, and then progressively decreased until it was almost absent in adults 36. It is reported that protein kinase A (PKA)-induced NF-κB/p-65 pathway activation facilitates SCs differentiation into a myelinating phenotype 37. Controversially, Morton showed that inhibition of NF-κB signaling resulted in no obvious differences in the structure or quantity of myelinated axons 38. Besides, using a transgenic mouse model, Morton demonstrated that NF-κB activation in SCs is not indispensable for myelination *in vivo* 39. However, to date, the role of NF-κB activation in SCs in the context of PDAC has not been investigated. In the present study, we recognized for the first time, the presence of phospho‑NF‑κB/p-65 in SCs in tissue sections from patients with PDAC, and revealed a positive correlation between phospho‑NF‑κB/p‑65 and IL6 in SCs. Moreover, we demonstrated that IL1β secreted by tumor cells is required for NF-κB activation in SCs, which could be abolished by the addition of anti-IL1β antibodies to the culture medium of SCs. As a result, NF-κB activation leads to upregulated expression of several pro-inflammatory cytokines in SCs, such as IL6, which in return promotes pancreatic cancer cell migration and spread *in vivo* and *ex vivo*.

Our study has some limitations. On the one hand, the extent to which our findings regarding the secreted cytokines and signaling cascades in SCs can be generalized to non-tumoral SCs is unknown, because the cell type being used as “human Schwann cells” (sNF96.2) was derived from neurofibromatosis type-1 disease. On the other hand, future studies are required to investigate whether interfering with the crosstalk between pancreatic cancer cells and SCs by targeting IL6 signaling could benefit patients with PDAC because of the inherent drawbacks of our animal model.

In conclusion, the current study reveals a previously neglected role of the tumor-neuroglia interaction through paracrine signaling in accelerating tumor dissemination. Taking our results together with those of others studies 8,11,13,40, SCs within the TME should be considered as an active and non-negligible player in tumor initiation and progression. Although further studies are urgently needed to clarify the potential mechanism of SC-induced tumor initiation and development, targeting the tumor-neuroglia interaction holds promise for the treatment of PDAC.

## Supplementary Material

Supplementary figures and tables.

## Figures and Tables

**Table 1:** Correlation of intra-tumoral SC density with clinicopathological features in PDAC samples

Variables	Number of cases	Low GFAP^+^/ S100^+^ group	High GFAP^+^/ S100^+^ group	*p* value
**Age (years)**				
≤ 60	26	9 (11.3%)	17 (21.3%)	0.057
>60	54	31 (38.8%)	23 (28.8%)	
**Gender**				
Male	43	19 (23.8%)	24 (30%)	0.268
Female	37	21 (26.3%)	16 (20%)	
**Histological grade**				
Well	13	5 (6.3%)	8 (10%)	0.104
Moderate	49	23 (28.8%)	26 (32.5%)	
Poor	18	12 (15%)	6 (7.5%)	
**Lymph node metastasis**				
No	31	15 (18.8%)	16 (20%)	0.821
Yes	49	25 (31.3%)	24(30%)	
**Distant metastases**				
No	65	36 (45%)	29 (36.3%)	0.046^*^
Yes	15	4 (5%)	11 (13.8%)	
**TNM stage**				
Ⅰ-Ⅱ	57	28 (35%)	29 (36.3%)	0.808
Ⅲ-Ⅳ	23	12 (15%)	11 (13.8%)	
**Vascular invasion**				
No	56	33 (41.3%)	23 (28.8%)	0.014^*^
Yes	24	7 (8.8%)	17 (21.3%)	
**Perineural invasion**				
No	38	24 (30%)	14 (17.5%)	0.025^*^
Yes	42	16 (20%)	26 (32.5%)	

*Denotes statistical significance. *p < 0.05

**Table 2:** Univariate and multivariate analysis of different prognostic parameters in patients with PDAC

Variables	Univariate analysis				Multivariate analysis		
	HR	95% CI	*p* value		HR	95% CI	*p* value
Age	0.689	0.386-1.231	0.208				
Gender	1.083	0.576-2.036	0.804				
Histological grade (Well *vs.* Moderate or Poor)	0.786	0.469-1.317	0.36				
Lymph node metastasis	1.762	0.951--3.266	0.072				
Distant metastases	3.447	1.745-6.808	< 0.001^***^		1.004	0.312-3.234	0.994
TNM stage (Ⅲ-Ⅳ *vs.* Ⅰ-Ⅱ)	3.181	1.711-5.915	< 0.001^***^		3.373	1.185-9.601	0.023^*^
Vascular invasion	1.138	0.613-2.113	0.681				
Perineural invasion	0.723	0.405-1.291	0.273				
Intra-tumor GFAP^+^S100^+^ (High vs. low)	1.867	1.032-3.38	0.039^*^		2.015	1.038-3.911	0.038^*^

Abbreviations: HR = hazard ratio; 95% CI = 95% confidence interval; *p < 0.05, ** *p*< 0.01, ***p < 0.001.

## Abbreviations

PDAC: pancreatic ductal adenocarcinoma
PAAD: pancreatic adenocarcinoma
PCa: pancreatic cancer
SC: Schwann cell
EMT: epithelia-mesenchymal transition
IL6: Interleukin 6
STAT3: signal transducer and activator of transcription 3
IL1β: Interleukin 1β
NF-κB: nuclear factor of kappa B
SCM: Schwann cells conditioned medium
TCM: tumor conditioned medium
Co-CM: co-cultured conditioned medium
TME: tumor microenvironment
PNS: peripheral nervous system
PANR: PDAC-associated neural remodeling
PNI: perineural invasion
