# Supplementary material for: Tumor-neuroglia interaction promotes pancreatic cancer metastasis
Source: Theranostics. 2020 Apr 6;10(11):5029–47. doi: 10.7150/thno.42440 (PMC7163446; doi:10.7150/thno.42440)
Supplement: Supplementary file 1 — Supplementary figures and tables. [file thnov10p5029s1.pdf]

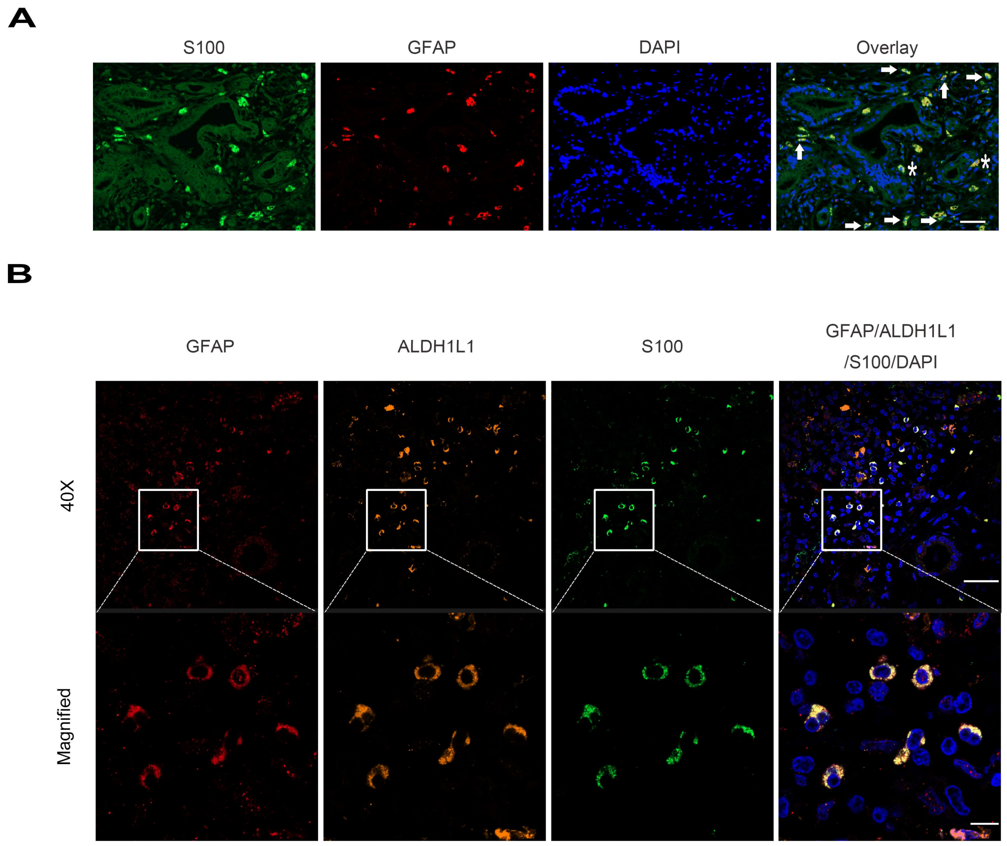

Figure S1. Schwann cells are located in PDAC stroma and directly contact with epithelial cells.

A. Representative double immunolabeling fluorescent images showing that Schwann cells are present in PDAC tissues. Schwann cells were found to be distributed in the PDAC stroma (white arrows). A subset of SCs were observed to closely contact with cancer cells (white asterisks). Scale bar: 50 μm.

B. PDAC tissue sections were immunolabeled with GFAP (red), ALDH1L1 (orange), S100 (green), and DAPI (blue). Images were acquired using a confocal microscope, demonstrating that cells detected via these markers were highly overlapped. Scale bar: 40 ×; 50 μm, Magnified view; 10 μm.

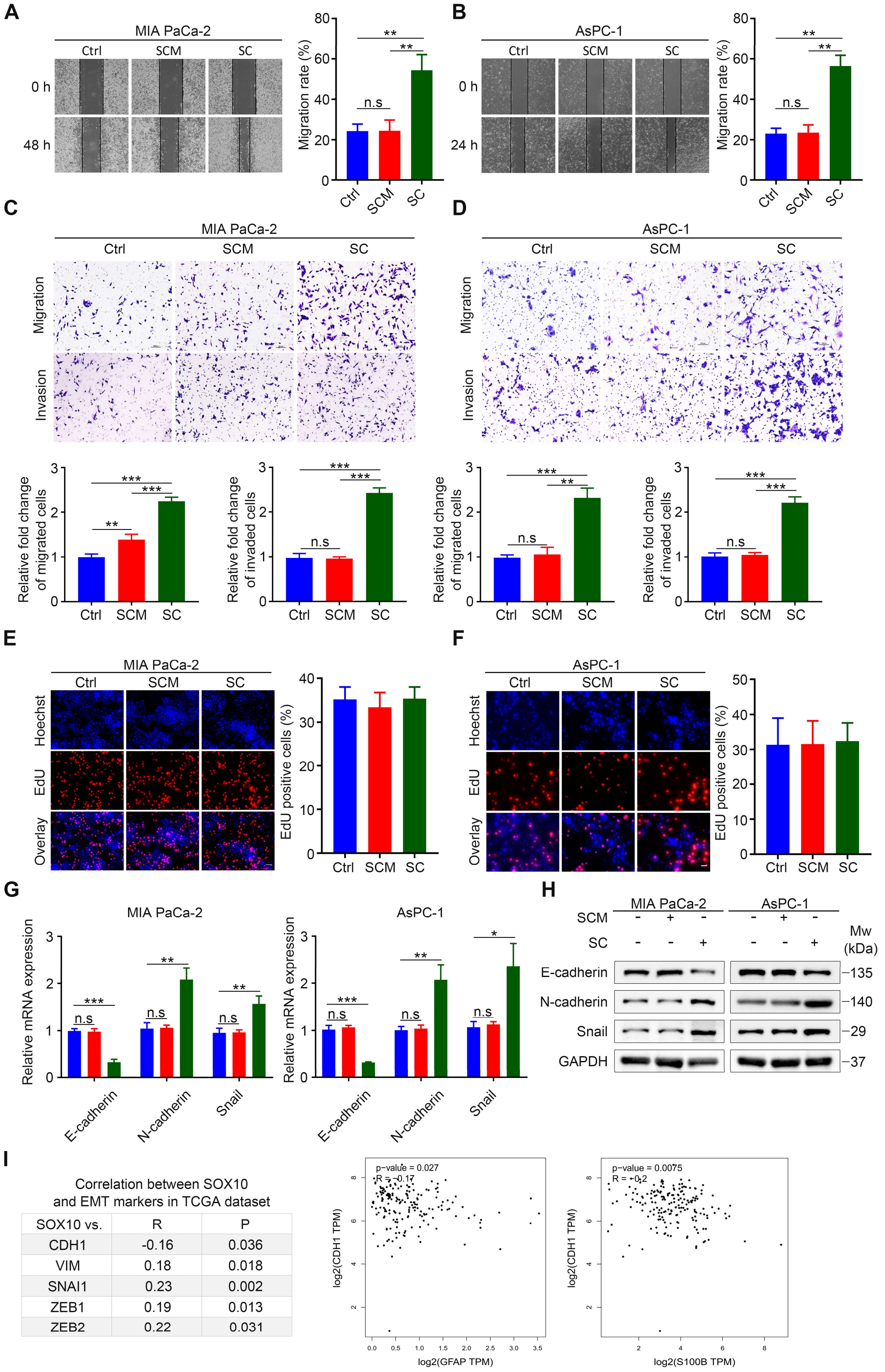

SOX10 vs.	R	P
CDH1	-0.16	0.036
VIM	0.18	0.018
SNAI1	0.23	0.002
ZEB1	0.19	0.013
ZEB2	0.22	0.031

Figure S2. Schwann cells increase the invasiveness of pancreatic cells and induce EMT *in vitro*. A and B. Representative images of a wound healing assay showing that rat SCs co-culture enhances the migration of MIA PaCa-2 and AsPC-1 cells, while rat Schwann cells conditioned medium (SCM) has no effect on the cell motility of MIA PaCa-2 and AsPC-1 cells. Medium supplemented with 1% FBS was used as a control. **C and D**. Representative images of Transwell migration and invasion assay showing that rat SCs co-culture enhances the migration and invasion of MIA PaCa-2 and AsPC-1 cells, whereas rat SCM has no such effects on MIA PaCa-2 and AsPC-1 cells. Medium containing 1% FBS was used as a control. Scale bar: 100 μm. **E and F**. Representative images of EdU proliferation assay showing neither rat SC co-culture nor rat SCM affected the proliferation of MIA PaCa-2 and AsPC-1 cells *in vitro*. Scale bar: 50 μm. **G**. qRT-PCR analyses showing that only rat SC co-culture could downregulate E-cadherin and upregulate the expression of N-cadherin and the epithelial-mesenchyme transition (EMT) regulator Snail simultaneously, indicating EMT of pancreatic cancer cells after co-cultivation with rat SCs. Cancer cells were cultured in control medium (3% FBS/DMEM) or 70% rat SCM or co-cultured with rat SCs for 24 h **H**. Immunoblotting analysis of E-cadherin, N-cadherin, and Snail levels in response to rat SCM incubation or rat SCs co-culture for 48 h. Cells cultured in control medium were used as a control. Loading control: GAPDH. **I**. Correlations between three Schwann cells markers (GFAP, S100, and SOX10) and EMT markers (CDH1, VIM, SNAI1, ZEB1, and ZEB2) in PDAC were acquired using the publicly available GEPIA tool. $^{*}p < 0.05$, $^{**}p < 0.01$, $^{***}p < 0.001$.

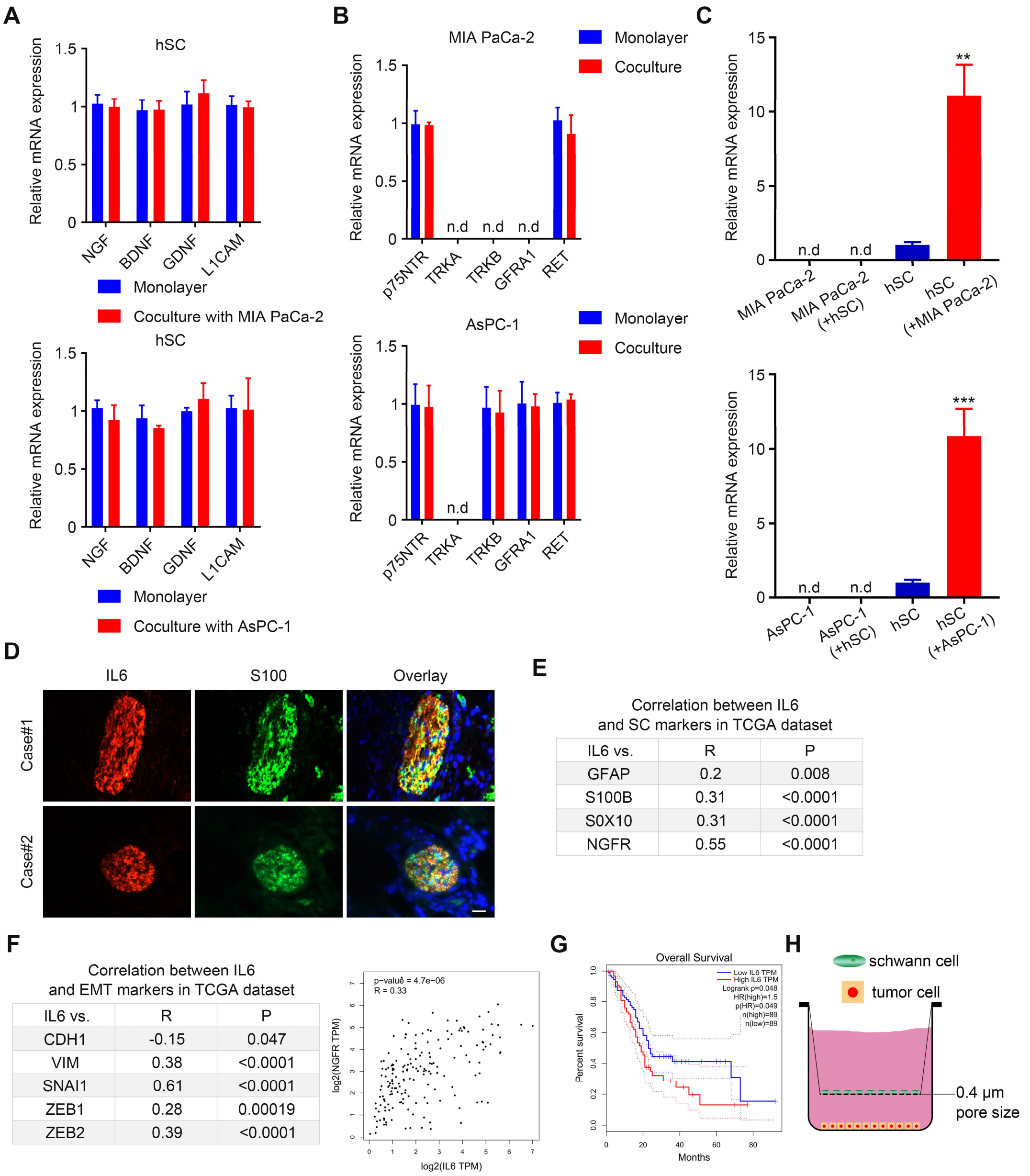

IL6 vs.	R	P
GFAP	0.2	0.008
S100B	0.31	<0.0001
S0X10	0.31	<0.0001
NGFR	0.55	<0.0001

IL6 vs.	R	P
CDH1	-0.15	0.047
VIM	0.38	<0.0001
SNAI1	0.61	<0.0001
ZEB1	0.28	0.00019
ZEB2	0.39	<0.0001

Figure S3. Schwann cells secret IL6 to promote pancreatic cell migration, invasion, and Epithelial-Mesenchymal transition *in vitro*. **A**. QRT-PCR analysis of neurotrophic factors *NGF*, *BDNF*, *GDNF*, and *L1CAM* in human SCs cultured in monolayers or co-cultured with pancreatic cancer cells. **B**. The expression of corresponding receptors in pancreatic cancer cells (*p75NTR*, *TRKA*, *TRKB*, *GFRA1*, and *RET*) cultured in monolayers or co-cultured with human SCs was assessed using qRT-PCR. **C**. QRT-PCR analysis of *IL6* mRNA level in SCs and pancreatic cancer cells (MIA PaCa-2 and AsPC-1) either cultured in monolayers or co-cultured with each other. **D**. Double immunolabeling fluorescent images showing intensive IL6 staining in S100-positive SCs in PDAC samples. Scale bar, 20 μm. **E**. Correlations between the gene expression of *IL6* and SC markers (*GFAP*, *S100B*, *SOX10*, *NGF*R) in PDAC were obtained using the publicly accessible cBioportal tool (TCGA PanCancer Atlas). **F**. Left chart: correlations between IL6 and EMT

markers (CDH1, VIM, SNAI1, ZEB1, and ZEB2) in PDAC were obtained using the publicly available GEPIA tool. Right chart: correlation between IL6 and SCs markers (NGFR) was analyzed using the publicly accessible GEPIA tool. **G**. Kaplan–Meier survival analysis was performed by comparing IL6-high pancreatic cancers (n = 89) and IL6-low pancreatic cancers (n = 89) using the GEPIA tool. **H**. Schematic illustration of the co-culture system. *$p < 0.05$, **$p < 0.01$, ***$p < 0.001$.

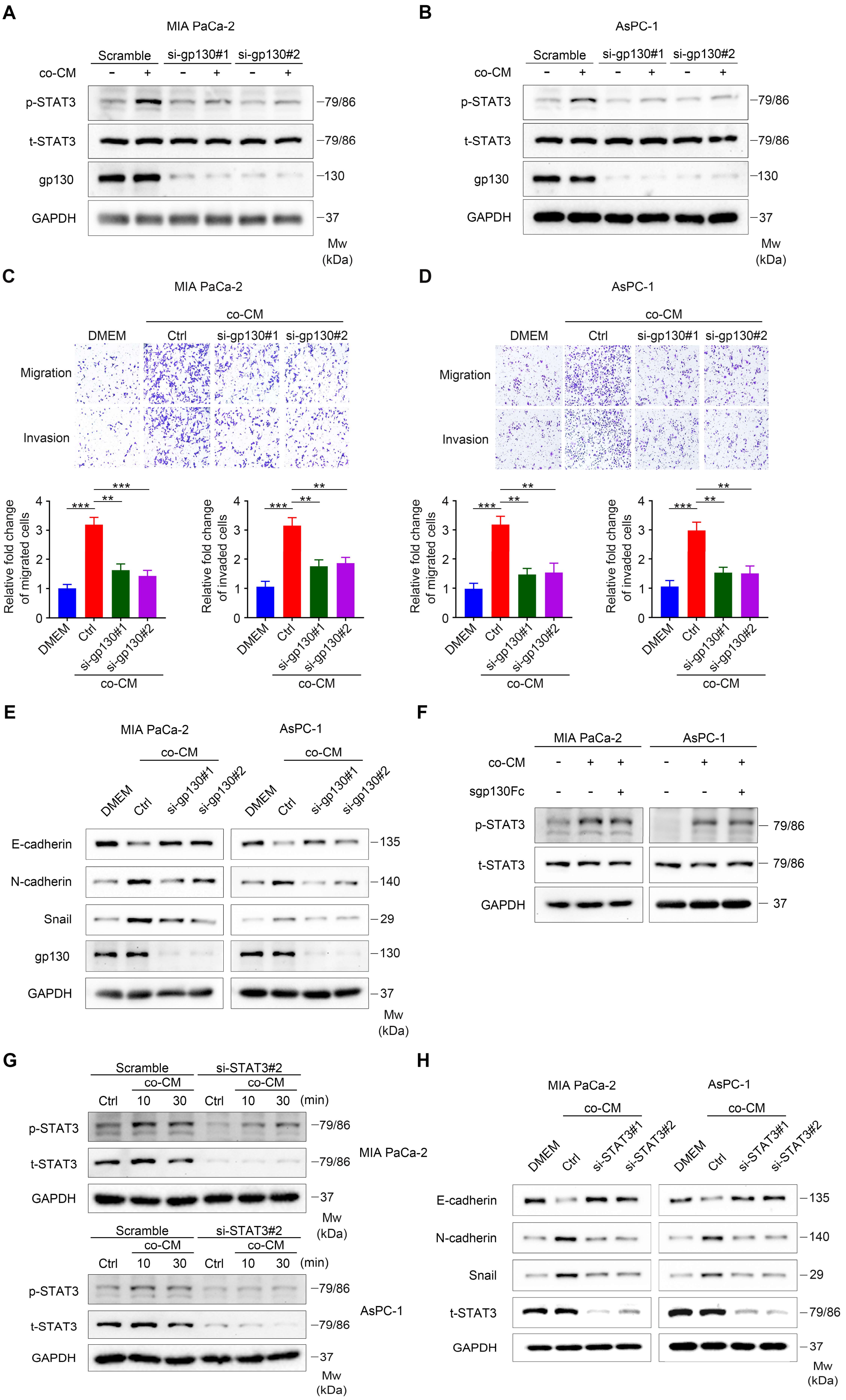

Figure S4. STAT3 signaling is critical for SC-induced pancreatic cancer cell migration and

invasion.

A and B. Scrambled siRNA or two gp130-specific siRNAs transfected MIA PaCa-2 or AsPC-1 cells were incubated in the absence or presence of co-CM for 30 min, after which cells were lysed with lysis buffer to detect STAT3 activation using immunoblotting. Loading control: GAPDH. **C and D.** Representative images of Transwell assays showing that in comparison with Scrambled siRNA-transfected MIA PaCa-2 or AsPC-1 cells, gp130-specific siRNAs-transfected tumor cells exhibited impaired pancreatic cancer cells locomotion and invasion. Control siRNA-transfected tumor cells incubated with 1% FBS/DMEM were used as a control. **E.** Scrambled siRNA or two gp130-specific siRNAs transfected MIA PaCa-2 or AsPC-1 cells were cultured in 70% co-CM (co-CM:10% FBS/DMEM) for 48 h, after which the cells were lysed for immunoblotting analysis. Scrambled siRNA-transfected cells grown in control medium (3% FBS/DMEM) were used as a control. Loading control: GAPDH. **F**. Cells were exposed to FBS-free medium, co-CM or co-CM plus 10 μg/ml sgp130Fc for 30 min and then collected for immunoblotting analysis. Loading control: GAPDH. **G.** Immunoblotting analysis showing disrupted STAT3 activation in MIA PaCa-2 or AsPC-1 cells following co-CM incubation at the indicated times in STAT3 siRNA#2-transfected MIA PaCa-2 or AsPC-1 cells. Scrambled siRNA-transfected MIA PaCa-2 or AsPC-1 cells exposed to co-CM were used as a control. Loading control: GAPDH. **H.** Immunoblotting analysis showing that STAT3 downregulation remarkably impaired co-CM induced EMT process of pancreatic cancer cells. Loading control: GAPDH.

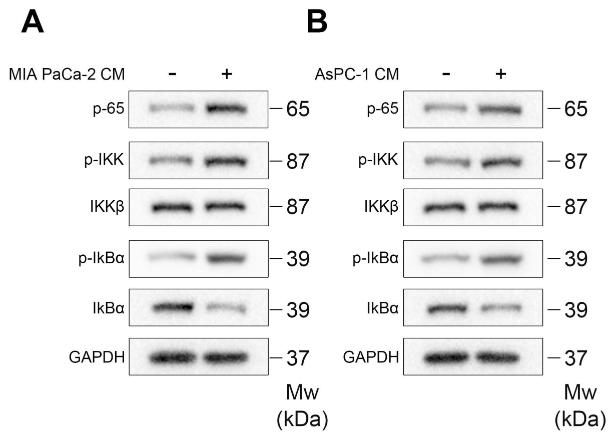

Fig S5. TCM treatment resulted in canonical NF-κB pathway activation.

A. Western blotting results showing activation of p65 in SCs upon addition of TCM derived from MIA PaCa-2 cells, as shown by the increased level of phospho-p65, paralleled to phosphorylation of IKK and IκBα, which was followed by its degradation. Loading control: GAPDH.

B. Western blotting results showing that activation of p65 in SCs upon addition of TCM derived from AsPC-1 cells, as shown by increased level of phospho-p65, paralleled to phosphorylation of IKK and IκBα, which was followed by its degradation. Loading control: GAPDH.

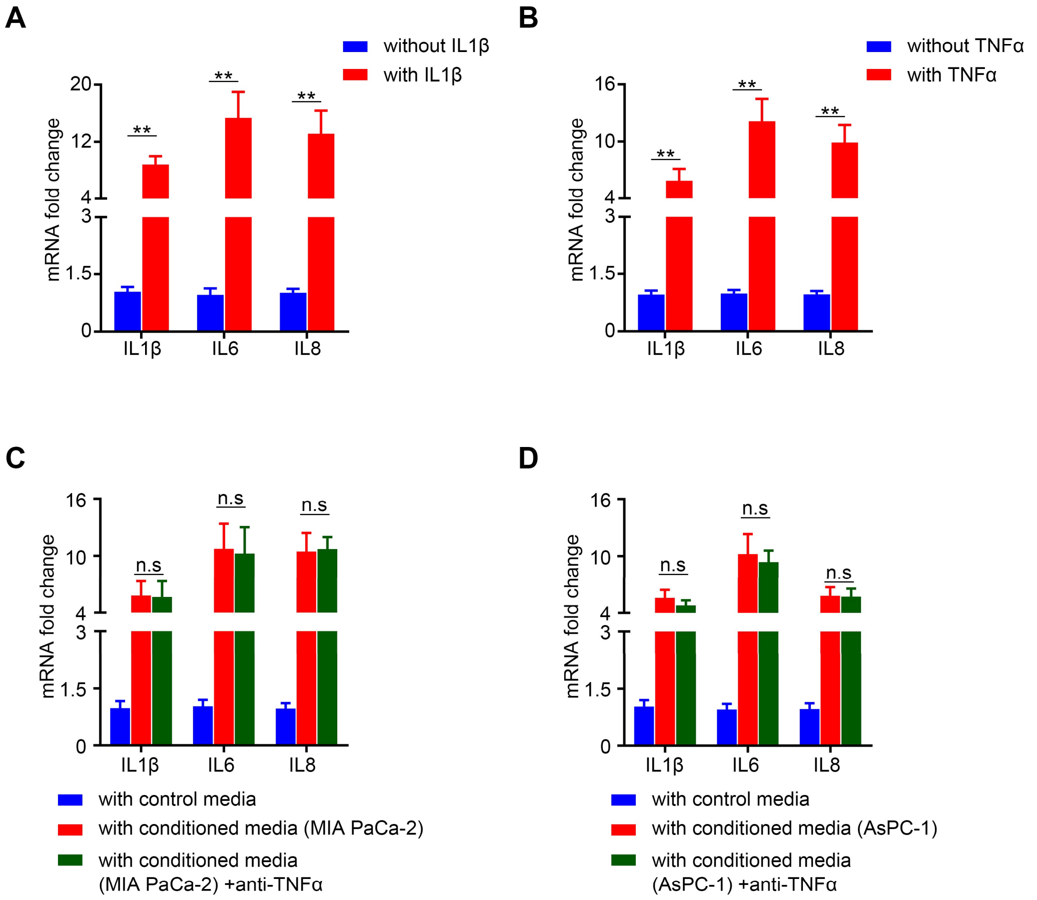

Fig S6. TNFα derived from pancreatic cells is not responsible for upregulated pro-inflammatory cytokines production from SCs.

A. Human Schwann cells were cultured in the absence or presence of 5 ng/ml recombinant human IL1β for 24 h, after which total RNA was extracted and subjected to qRT-PCR analysis.

B. Human Schwann cells were cultured without or with 10 ng/ml recombinant human TNFα for 24 h, after which total RNA was extracted and subjected to qRT-PCR analysis.

C. QRT-PCR analysis of *IL1B*, *IL6*, and *IL8* mRNA levels in human SCs cultured in control medium or 70% TCM derived from MIA PaCa-2 cells in the absence or presence of 20 ng/ml TNFα neutralizing antibody for 24 h.

D. RT-qPCR analysis of *IL1B*, *IL6*, and *IL8* mRNA level in human SCs cultured in control medium or 70% TCM derived from AsPC-1 cells in the absence or presence of 20 ng/ml TNFα neutralizing antibody for 24 h.

Table S1. Primer sequence for qRT-PCR

Gene	Forward primer (5′ to 3′)	Reverse primer (5′ to 3′)
**E-cadherin**	CGAGAGCTACACGTTCACGG	GGGTGTCGAGGGAAAAATAGG
**N-cadherin**	AGCCAACCTTAACTGAGGAGT	GGCAAGTTGATTGGAGGGATG
**Snail**	TCTTTCCTCGTCAGGAAGC	AGGTAAACTCTGGATTAGAGTCC
**TNF**	GAGGCCAAGCCCTGGTATG	CGGGCCGATTGATCTCAGC
**IL1A**	TGGTAGTAGCAACCAACGGGA	ACTTTGATTGAGGGCGTCATTC
**IL1B**	ACGATGCACCTGTACGATCA	TCTTTCAACACGCAGGACAG
**IL6**	ACTCACCTCTTCAGAACGAATTG	CCATCTTTGGAAGGTTCAGGTTG
**CXCL8**	ACTGAGAGTGATTGAGAGTGGAC	AACCCTCTGCACCCAGTTTTC
**LIF**	CCAACGTGACGGACTTCCC	TACACGACTATGCGGTACAGC
**CCL2**	CAGCCAGATGCAATCAATGCC	TGGAATCCTGAACCCACTTCT
**IL1R1**	ATGAAATTGATGTTCGTCCCTGT	ACCACGCAATAGTAATGTCCTG
**IL6R**	CCCCTCAGCAATGTTGTTTGT	CTCCGGGACTGCTAACTGG
**GP130**	CGGACAGCTTGAACAGAATGT	ACCATCCCACTCACACCTCA
**CXCR1**	GGCCGGTGCTTCAGTTAGAT	GGGCATAGGCGATGATCACA
**CXCR2**	CCTGTCTTACTTTTCCGAAGGAC	TTGCTGTATTGTTGCCCATGT
**NGF**	TGTGGGTTGGGGATAAGACCA	GCTGTCAACGGGATTTGGGT
**BDNF**	ATTGGCTGGCGATTCATAAG	GTTTCCCTTCTGGTCATGGA
**GDNF**	TGACTCCAATATGCCTGAAGATTATC	AATGGTGGCTTGAATAAAATCCA
**L1CAM**	TGTCATCACGGAACAGTCTCC	CTGGCAAAGCAGCGGTAGAT
**p75NTR**	CCGTTGGATTACACGGTCCAC	TGAAGGCTATGTAGGCCACAA
**TRKA**	AACCTCACCATCGTGAAGAGT	TGAAGGAGAGATTCAGGCGAC
**TRKB**	TCGTGGCATTTCCGAGATTGG	TCGTCAGTTTGTTTCGGGTAAA
**GFRA1**	CCAAGCACAGCTACGGAATG	CAGGCACGATGGTCTGTCG
**RET**	ACACGGCTGCATGAGAACAA	GCCCTCACGAAGGGATGTG
**GAPDH**	GCTAAGCAGTTGGTGGTGCA	TCACCACCATGGAGAAGGC

Table S2. siRNA sequences for cell transfection

Gene	Sense (5'→3')	Antisense (5'→3')
**Negative control**	5'-UUCUCCGAACGUGUCACGUTT-3'	5'-ACGUGACACGUUCGGAGAATT-3'
**siSTAT3#1**	5'-CCCGUCAACAAAUUAAGAATT-3'	5'-UUCUUAAUUUGUUGACGGGTT-3'
**siSTAT3#2**	5'-GCAACAGAUUGCCUGCAUUTT-3'	5'-AAUGCAGGCAAUCUGUUGCTT-3'
**siIL1R1#1**	5'-GGAAAGACCUAUGACGCAUTT-3'	5'-AUGCGUCAUAGGUCUUUCCTT-3'
**siIL1R1#2**	5'-CCUCAUCACAGUGCUUAAUTT-3'	5'-AUUAAGCACUGUGAUGAGGTT-3'
**siIL1B#1**	5'-GCCAGGAUAUAACUGACUUTT-3'	5'-AAGUCAGUUAUAUCCUGGCTT-3'
**siIL1B#2**	5'-GGUGAUGUCUGGUCCAUAUTT-3'	5'-AUAUGGACCAGACAUCACCTT-3'
**siGP130#1**	5'-GCAUCCAGUGUCACCUUUATT-3'	5'-UAAAGGUGACACUGGAUGCTT-3'
**siGP130#2**	5'-GCACCAAGUUUCUGGUAUATT-3'	5'-UAUACCAGAAACUUGGUGCTT-3'

Table S3. Primary and secondary antibodies

Antibody	Species	Type	Dilution	Source
**GFAP**	Rabbit	Polyclonal	1:400 (IF)	Dako
**GFAP**	Goat	Polyclonal	1:100 (IF)	Abcam
**S100**	Mouse	Monoclonal	1:500 (IHC),1:50 (IF)	Abcam
**S100**	Rabbit	Monoclonal	1:100 (IF)	Abcam
**ALDH1L1**	Mouse	Monoclonal	1:40 (IF)	Abcam
**E-cadherin**	Mouse	Monoclonal	1:1000 (WB)	Cell Signaling
**N-cadherin**	Rabbit	Monoclonal	1:1000 (WB)	Cell Signaling
**Snail**	Rabbit	Monoclonal	1:1000 (WB)	Cell Signaling
**GAPDH**	Mouse	Monoclonal	1:5000 (WB)	Ray Antibody
**IL1R1**	Rabbit	Polyclonal	1:1000 (WB)	Abcam
**phospho-STAT3**	Rabbit	Monoclonal	1:2000 (WB), IF (1:100)	Cell Signaling
**t-STAT3**	Mouse	Monoclonal	1:1000 (WB)	Cell Signaling
**gp130**	Rabbit	Monoclonal	1:1000 (WB)	Cell Signaling
**phospho-NF-κB/p65 (Ser536)**	Rabbit	Monoclonal	1:1000 (WB)	Cell Signaling
**NF-κB/p65**	Rabbit	Monoclonal	1:1000 (WB)	Cell Signaling
**Phospho-IKKα/β (Ser176/180)**	Rabbit	Monoclonal	1:1000 (WB)	Cell Signaling
**IKKβ**	Rabbit	Monoclonal	1:1000 (WB)	Cell Signaling
**IκBα**	Mouse	Monoclonal	1:1000 (WB)	Cell Signaling
**Phospho-IκBα (Ser32)**	Rabbit	Monoclonal	1:1000 (WB)	Cell Signaling
**Phosphor-NF-kB/p65 (S536)**	Rabbit	Polyclonal	1:100 (IHC)	Abcam
**IL6**	Rabbit	Polyclonal	1:200 (IHC),1:50 (IF)	Abcam
**Alexa Fluor 555 donkey anti-rabbit IgG**	Donkey	Polyclonal	1:300 (IF)	Abcam
**Alexa Fluor 488 donkey anti-mouse IgG**	Donkey	Polyclonal	1:300 (IF)	Abcam
**Alexa Fluor 647 donkey anti-goat IgG**	Donkey	Polyclonal	1:300 (IF)	Abcam

IF, immunofluorescence; IHC, immunohistochemistry; WB, Western blotting.
